# Mucosal Administration of Cycle-Di-Nucleotide-Adjuvanted Virosomes Efficiently Induces Protection against Influenza H5N1 in Mice

**DOI:** 10.3389/fimmu.2017.01223

**Published:** 2017-09-28

**Authors:** Thomas Ebensen, Jennifer Debarry, Gabriel K. Pedersen, Paulina Blazejewska, Sebastian Weissmann, Kai Schulze, Kenneth C. McCullough, Rebecca J. Cox, Carlos A. Guzmán

**Affiliations:** ^1^Department of Vaccinology and Applied Microbiology, Helmholtz Centre for Infection Research, Braunschweig, Germany; ^2^The Influenza Centre, University of Bergen, Bergen, Norway; ^3^Jebsen Centre for Influenza Vaccine Research, Department of Clinical Science, University of Bergen, Bergen, Norway; ^4^Department of Research and Development, Haukeland University Hospital, Bergen, Norway; ^5^Institute of Virology and Immunology, Mittelhäusern, Switzerland

**Keywords:** mucosal adjuvant, cyclic di-adenosine monophosphate, sublingual administration, c-di-GMP, dose-sparing capacity

## Abstract

The need for more effective influenza vaccines is highlighted by the emergence of novel influenza strains, which can lead to new pandemics. There is a growing population of susceptible subjects at risk for severe complications of influenza, such as the elderly who are only in part protected by current licensed seasonal vaccines. One strategy for improving seasonal and pandemic vaccines takes advantage of adjuvants to boost and modulate evoked immune responses. In this study, we examined the capacity of the recently described adjuvant cyclic di-adenosine monophosphate (c-di-AMP) to serve as an adjuvant for improved mucosal influenza vaccines, and induce effective protection against influenza H5N1. In detail, c-di-AMP promoted (i) effective local and systemic humoral immune responses, including protective hemagglutination inhibition titers, (ii) effective cellular responses, including multifunctional T cell activity, (iii) induction of long-lasting immunity, and (iv) protection against viral challenge. Furthermore, we demonstrated the dose-sparing capacity of the adjuvant as well as the ability to evoke cross-clade protective immune responses. Overall, our results suggest that c-di-AMP contributes to the generation of a protective cell-mediated immune response required for efficacious vaccination against influenza, which supports the further development of c-di-AMP as an adjuvant for seasonal and pandemic influenza mucosal vaccines.

## Introduction

Seasonal and pandemic influenza remain major causes of severe morbidity and mortality worldwide and cause huge economic loss (1). While vaccination is the cornerstone of influenza prophylaxis, seasonal vaccines provide only partial protection, especially in high-risk groups (2). Novel highly pathogenic avian influenza virus strains A H5N5 and H5N8 viruses caused outbreaks in Asia, Europe, and North America and the novel type A influenza virus A (H7N9) strain emerging in China—increase concern over the threat of a new pandemic (3). In this regard, the global spread within 2 months of the 2009 H1N1 virus pandemic showed clearly that current vaccine platforms cannot meet requirements during an emerging pandemic (4). The global vaccine manufacturing capacity was inadequate and unable to supply enough vaccine doses in a timely manner (5, 6).

To date, most of the licensed seasonal vaccines are administered *via* parenteral routes; while inducing sufficient systemic immune responses, they fail to promote mucosal immunity (7). This is an important issue considering that the respiratory tract mucosa is the entry portal for influenza viruses. Mucosal immunization offers the potential for protective responses at both the systemic and local level, e.g., by induction of secretory IgA and cytotoxic T lymphocyte (CTL) activity (8). Although current (split and subunit) vaccines are considered very safe, they do not elicit adequate immune responses when administered *via* mucosal routes. Moreover, no mucosal adjuvant has been licensed worldwide so far. This can be related to either a lack of activity—the widely used alum does not provide mucosal adjuvant activity—or to safety concerns—the cholera toxin subunit B (CTB) adjuvant was transported to the nervous system *via* olfactory nerves when applied intranasally (i.n.), and an influenza vaccine containing *Escherichia coli* heat-labile enterotoxin (LT) was linked to Bell’s palsy (9). Nevertheless, new promising candidates, such as members of the cyclic-di-nucleotide family among others, are under development (10, 11). The cyclic-di-nucleotides are second-messenger molecules in bacteria and archea (12, 13) and the innate immune system senses them *via* STING (stimulator of interferon genes) and DDX41 [DEAD (aspartate-glutamate-alanine-aspartate)-box helicase 41] (14, 15). In addition, these molecules can stimulate IL-1β secretion through the NLRP3 inflammasome, which is independent of STING (16). Not surprisingly, therefore, these cyclic-di-nucleotides possess adjuvant properties, promoting the development of local and systemic immune responses when administered by different routes (17–23) and stimulate a balanced Th cell response (17, 18), which would be important for anti-influenza immunity (19–21, 24).

However, the severe complications associated with i.n. vaccination have provoked safety concerns arguing against this strategy for mass mucosal vaccination. An alternative administration route would be *via* the sublingual (s.l.) mucosa, which provides several advantages—formulations are easy to administer, the route has no risk of cross-contamination, and s.l. vaccination would be highly accepted due to the needle-free application (8, 25). Moreover, s.l. vaccination can be delivered by personnel without medical training, and the route is already well established as s.l. immunotherapy (SLIT) targeting allergies, with which no anaphylactic shock or other adverse side effects have been observed in human studies (26). Due to the compartmentalization of the mucosal immune system, the resulting responses are induced at both the site of administration and at distant sites (e.g., nose and vagina) (8).

Approval of influenza vaccines in Europe requires demonstration of a protective serological hemagglutination inhibition (HAI) titer above 40, yet other immunologic correlates are also important—induction of cellular responses, especially in high-risk groups and effective immune memory (27). In this context, an important aspect of adjuvants is the ability to modulate and potentiate immune responses (17, 18). Adjuvanted influenza vaccines also permit dose sparing and the potential for reducing the number of booster vaccinations (19–21, 24, 28).

In this study, we examined the capacity of the recently described cyclic di-adenosine monophosphate (c-di-AMP) adjuvant to induce effective protection against influenza H5N1 by mucosal vaccination, together with the potential for enabling dose sparing. We demonstrated that c-di-AMP promotes the induction of a protective immune response in mice against influenza H5N1 (A/Vietnam/1194/2004, NIBRG-14) when administered by i.n. as well as s.l. route. Efficacy at inducing both local and systemic immune responses was observed, which included protective HAI titers, efficient Th cell responses, and a long-lasting immune response. Furthermore, application of the c-di-AMP facilitated dose sparing and cross-clade reactive immune responses against drifted strains, such as A/Anhui/1/2005 (IBCDC-RG6). Together, this supports the further development of c-di-AMP as an adjuvant for seasonal and pandemic influenza mucosal vaccines.

## Materials and Methods

Information that is more detailed is provided in Methods in Supplementary Material available online only.

### Animals

Six- to eight-week-old female BALB/c mice were purchased from Harlan Winkelmann (Germany) or Janvier Labs (France) and maintained in the animal care facility of the Helmholtz Centre for Infection Research. All animal experiments were approved by the institutional ethical board and conducted in accordance to the regulations of the local government of Lower Saxony (Germany; No. 509.42502-04-017.08).

### Immunization Protocols

Groups of mice (three to five animals) were immunized either i.n. or s.l. on days 0 and 21 with PBS or isotonic saline (mock), or with H5N1 virosomes; the latter were administered alone or with different adjuvants—c-di-AMP, c-di-GMP, CTB (Sigma-Aldrich, Germany), last (or third) generation adjuvant (LGA) of immune stimulating complexes (Matrix M™)—made up to a maximal volume of 20 µl (i.n.) or 10 µl (s.l.) in PBS or isotonic saline solution.

### Sample Collection and Processing

Blood samples were collected on days 1, 20, and 42 *via* retro-bulbar bleeding. On day 42, saliva was collected prior to the blood sample by temporarily anesthetizing animals by i.p. injection of Ketamine/Rompun. Then, mice were sacrificed and broncho-alveolar and nasal lavages were obtained by flushing the local tissues with ice-cold PBS.

### Virus and Mouse Challenge Studies

The influenza virosomal vaccine (Crucell, Netherlands) was produced from the reverse genetics-engineered seed virus (NIBRG-14), which was derived from a re-assortment between A/Vietnam/1194/2004 (H5N1) and A/Puerto Rico/8/34 (H1N1) (NIBSC, UK). These H5N1 virosomes contain the surface hemagglutinin (HA) and neuraminidase proteins embedded in a lipid membrane with no internal proteins. For challenge experiments, mice were anesthetized by i.p. injection with Ketamine/Rompun and challenged with 2 × 10^3^ focus forming units (ffu) of influenza H5N1 (A/Vietnam/1194/2004, NIBRG-14) virus given by i.n. route.

### Detection of Antigen-Specific IgG and IgA in Serum

The H5N1-specific antibodies were determined in serum samples by ELISA. Endpoint titers were expressed as reciprocal values of the last dilution, which gave an optical density (OD) at 405 nm of two times above the values of the negative controls. For calculation purposes, negative samples were assigned an arbitrary titer of the lowest dilution measured.

### Determination of Antigen-Specific IgA

The amount of total and H5N1-specific IgA present in the lavages was determined by ELISA. To compensate for variations among animals in the recovery efficiency of secretory antibodies, the results were normalized and expressed as endpoint titers of antigen-specific IgA per μg of total IgA present in the sample. For calculation purposes, samples negative for specific IgA were assigned an arbitrary titer of the lowest dilution measured.

### Determination of Total IgE

The content of total IgE in sera was determined using an anti-mouse IgE ELISA Kit (ELISA MAX™ Deluxe Set, BioLegend, USA) according to the manufacturer’s instructions. Endpoint titers were expressed as absolute values of the last dilution that gave an OD at 405 nm of two times above the values of the negative controls.

### Measurement of Cellular Proliferation

Spleens of vaccinated mice were aseptically removed. For the subsequent methods, cell suspensions of spleens (*n* = 5) of each immunized groups were prepared and erythrocytes were lysed. These splenocyte pools of each group were cultured in the presence of different concentrations of inactivated NIBRG-14 virus with an HA concentration of 0.1–4 µg/mL; controls received 5 µg/mL concanavalin A. The incorporation of [^3^H] thymidine into the DNA of proliferating cells was determined using a scintillation counter (Wallac 1450, Micro-Trilux).

### ELISpot Assay

In order to determine the number of cytokine-secreting cells in the spleen, murine IFN-γ, IL-2, IL-4, and IL-17 ELISpot kits (BD Pharmingen) were used according to the manufacturer’s instructions. Colored spots were counted with an ELISpot reader (C.T.L.) and analyzed using the ImmunoSpot image analyzer software v3.2.

### Multiplex FlowCytomix [Cytometric Bead Array (CBA)]

The FlowCytomix immunoassay was used in order to quantify the cytokines and chemokines secreted by splenocytes restimulated ex vivo using inactivated NIBRG-14 virus (NIBSC) or H5N1 virosomes (Crucell, Netherlands). The presence of mouse Th1/Th2/Th9/Th17 cytokines was determined with a FlowCytomix immunoassay according to the manufacturer’s instructions (eBioscience).

### HAI Assay

The reciprocal of the serum dilution represents the HAI titer of the respective serum. Negative samples were assigned an arbitrary titer of 5 for calculation purposes.

### MN Assay

The MN assay has been shown to be more sensitive than the HAI assay for the measurement of humoral immunity against influenza viruses. The absorbance (OD) was measured at 490 nm.

### Cross-Clade Reactivity

Cross-reactive immune responses against the drifted strains A/Cambodia/R0405050/2007 (clade 1.1), A/turkey/Turkey/1/2005 (clade 2.2.1), and A/Anhui/1/2005 (clade 2.3.4) were analyzed using the HAI assay.

### Multifunctional T Cells

Splenocytes (2 × 10^7^ cells/mL) were incubated (37°C, 5% CO_2_) in RPMI containing the HA antigen (NIBRG-14, 200 ng HA/mL) or no antigen to determine the basal cytokine production. Viable singlet leukocytes were gated for CD3^+^CD4^+^CD8^−^ and subsequently analyzed for the expression of intracellular IL-2, IL-4, IL-17, TNF-α, and IFN-γ.

### Statistical Analysis

The statistical significance of the differences observed between the different experimental groups was analyzed using one-way ANOVA with Tukey’s or Dunnett’s *post hoc* test (GraphPad Prism v.6) with titers log_2_ normalized. Differences were considered significant at *p* < 0.05.

## Results

### c-di-AMP Promotes Systemic and Mucosal Antibody Responses against H5N1

BALB/c mice were vaccinated i.n. with H5N1 virosomes alone or adjuvanted with c-di-AMP. Negative controls received only PBS; positive controls received H5N1 virosomes with one of three adjuvants—CTB as gold standard for mucosal adjuvants, an LGA (Matrix M™), or another cyclic-di-nucleotide, c-di-GMP. The c-di-AMP adjuvant clearly promoted high systemic antibody titers of vaccine-induced antigen-specific IgG and IgG1 in the serum (Table 1).

**Table 1:** Systemic antibody response.

	Control	H5N1 alone	H5N1 + c-di-AMP	H5N1 + c-di-GMP	H5N1 + CTB	H5N1 + LGA
IgG	5.61E+02**** (4.92E+02, 6.41E+02)	4.17E+03**** (2.39E+03, 7.27E+03)	1.20E+06 (7.37E+05, 1.96E+06)	1.02E+06 (1.02E+06, 1.02E+06)	6.76E+05 (4.19E+05, 1.09E+06)	6.45E+05 (2.67E+05, 1.56E+06)

IgG1	7.94E+02**** (5.94E+02, 1.06E+03)	1.15E+04**** (4.82E+03, 2.77E+04)	2.28E+06 (1.63E+06, 3.19E+06)	5.12E+05 (9.15E+04, 2.87E+06)	2.05E+06 (1.07E+06, 3.92E+06)	3.57E+06 (1.65E+06, 7.69E+06)

IgG2a	7.64E+02**** (6.00E0+2, 9.71E+02)	2.08E+03**** (1.29E+03, 3.36E+03)	1.66E+06 (8.11E+05, 3.38E+06)	8.13E+05 (3.01E+05, 2.20E+06)	1.54E+05**** (7.51E+04, 3.16E+05)	6.86E+04**** (2.82E+04, 1.67E+05)

Ratio IgG2a/IgG1	0.94 ± 0.06	0.2 ± 0.09	0.86 ± 0.33	1.4 ± 0	0.09 ± 0.03*	0.02 ± 0.01*

IgG2b	7.07E+02**** (5.92E+02, 8.44E+02)	1.39E+03**** (7.76E+02, 2.47E+03)	1.20E+06 (6.97E+05, 2.07E+06)	1.02E+06 (1.02E+06, 1.02E+06)	3.85E+04**** (1.14E+04, 1.30E+05)	7.35E+04**** (2.90E+04, 1.86E+05)

IgG3	6.19E+02**** (5.06E+02, 7.57E+02)	6.53E+02**** (4.97E+02, 8.57E+02)	5.17E+04 (3.36E+04, 7.97E+04)	1.02E+05 (7.32E+03, 1.41E+06)	1.71E+04* (9.06E+03, 3.25E+04)	7.46E+03****(2.89E+03, 1.93E+04)

IgE	9.04E+02 (4.93E+02, 1.66E+03)	1.16E+03 (5.85E+02, 2.28E+03)	7.19E+02 (3.42E+02, 1.51E+03)	1.00E+02^+^ (1.00E+02, 1.00E+02)	4.22E+03^++^ (2.37E+02, 7.52E+03)	4.85E+03^++^(3.76E+03, 6.27E+03)

Groups of 3–5 BALB/c mice were immunized by i.n. route with PBS (control), or with two doses (21 days apart) of H5N1 virosomes (7.5 µg HA), alone or adjuvanted with c-di-AMP (5 µg), c-di-GMP (5 µg), CTB (10 µg), or LGA (7.5 µg). The number of independent experiments for all analyses except the IgE measurement was 1 for c-di-GMP, 2 for LGA, 3 for c-di-AMP and CTB, and 4 for the control and antigen alone; in the case of IgE measurement 1 for c-di-GMP, 2 for LGA and CTB, and 3 for c-di-AMP, the control and antigen alone. Influenza virus-specific serum IgG and subclasses (IgG1, IgG2a, IgG2b, and IgG3) and total IgE were measured by ELISA 21 days after the second immunization. The geometric mean of the endpoint titer is shown with the 95% CI in parentheses. The IgG2a/IgG1 ratio is shown as mean ± SEM (ratios >1 indicate a Th1 polarization and ratios <1 a Th2 polarization). Statistically significant differences were measured by one-way ANOVA with Tukey’s post hoc test of the log2 normalized data; differences from the c-di-AMP group or the negative control group are shown by asterisks or plus signs, respectively (*/^+^p < 0.05, **/^++^p < 0.01, ***/^+++^p < 0.001, ****/^++++^p < 0.0001).

These antibody IgG titers were comparable to those obtained with the other adjuvanted groups, and significantly higher than the levels with the negative control and antigen alone groups. High titers of antigen-specific IgG2a, IgG2b, and IgG3 were also detected in the adjuvanted groups, with the c-di-AMP-adjuvanted group showing significantly higher titers compared to those with CTB and LGA. The cyclic-di-nucleotide-adjuvanted group also showed a balanced IgG2a/IgG1 ratio comparable to that from the antigen alone group; CTB and LGA adjuvanting promoted a Th2 shift visualized by a low ratio (0.09 and 0.02, respectively).

**Figure 1:**
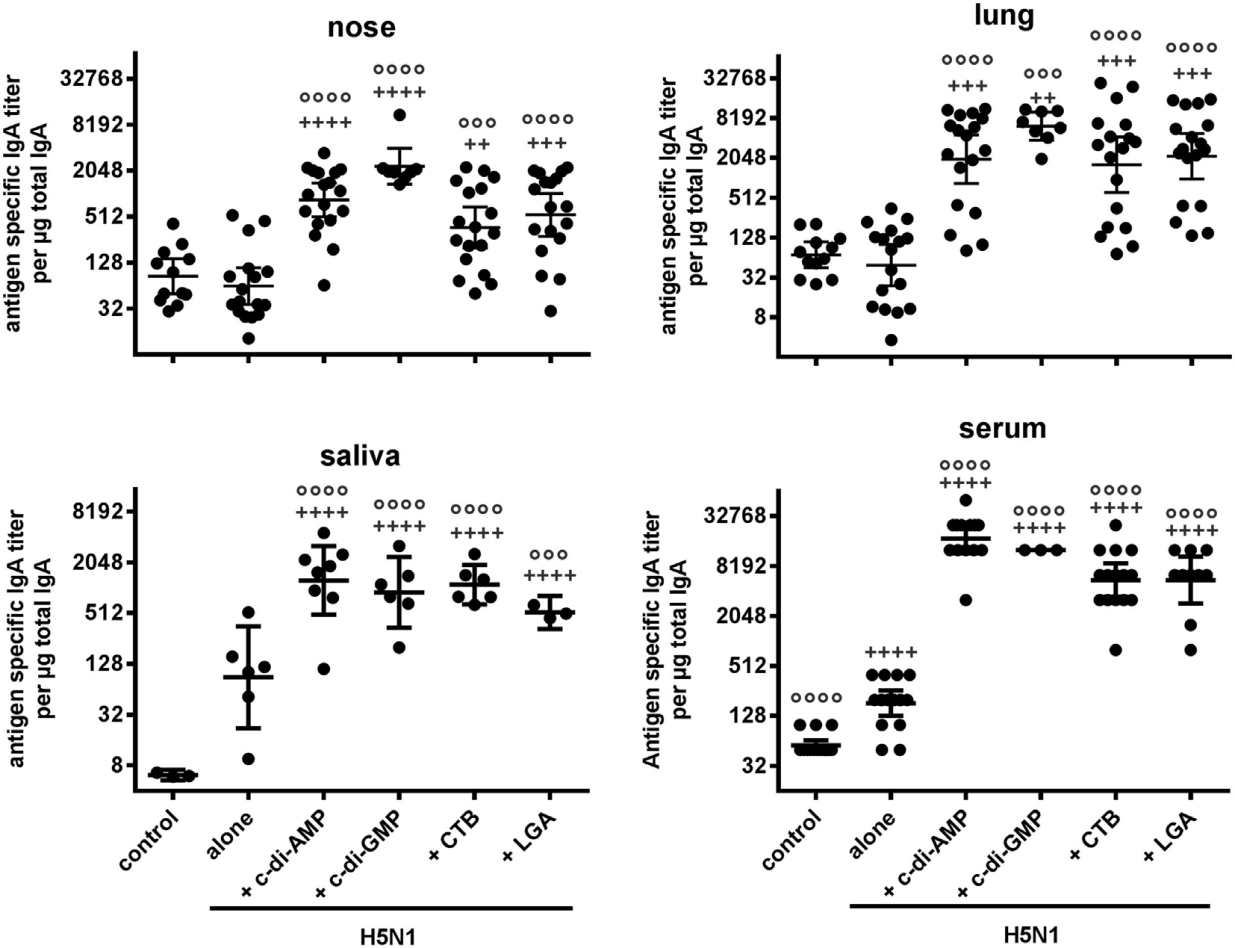
Mucosal antibody response. Groups of BALB/c mice were immunized intranasally with PBS (control), or with two doses (21 days apart) of H5N1 virosomes (7.5 µg hemagglutinin), alone or adjuvanted with cyclic di-adenosine monophosphate (c-di-AMP) (5 µg), c-di-GMP (5 µg), cholera toxin subunit B (CTB) (5 µg for the saliva experiment, or 10 µg), or last (or third) generation adjuvant (LGA) (7.5 µg). At 21 days after the last immunization, sera, saliva, lung, and nasal washes were collected. Total and antigen-specific IgA titers were measured by ELISA. The geometric mean with 95% CI of the endpoint titer of antigen-specific IgA per μg total IgA in the case of the lavages, and the endpoint titer of antigen-specific IgA in the case of the serum were obtained from *n* = 4 (alone), *n* = 3 (PBS, c-di-AMP, CTB), *n* = 2 (LGA), or *n* = 1 (c-di-GMP) independent experiments. Each symbol represents one animal. Statistically significant differences were measured by one-way ANOVA with Tukey’s *post hoc* test of the log_2_ normalized data; differences from the negative control or antigen alone group are shown by plus signs or circles, respectively (^+^/°*p* < 0.05, ^++^/°°*p* < 0.01, ^+++^/°°°*p* < 0.001, ^++++^/°°°°*p* < 0.0001).

In all adjuvanted groups, high antigen-specific serum IgA titers were also measured in saliva, nasal samples, and lung lavages, and were significantly increased compared to the antigen alone and negative control groups at distant local sites (Figure 1). Animals receiving CTB- and LGA-adjuvanted vaccine showed low nasal IgA titers, albeit still higher than those obtained with antigen alone (Figure 1). It is also important that immunization *via* mucosal routes does not induce enhanced IgE titers. Both reference controls with CTB- and LGA-adjuvanted vaccines showed only slightly increased total IgE titers compared with the antigen alone group, whereas c-di-AMP-adjuvanted vaccine did not show enhanced IgE titers (Table 1).

### c-di-AMP Promotes Cellular Responses to H5N1

**Figure 2:**
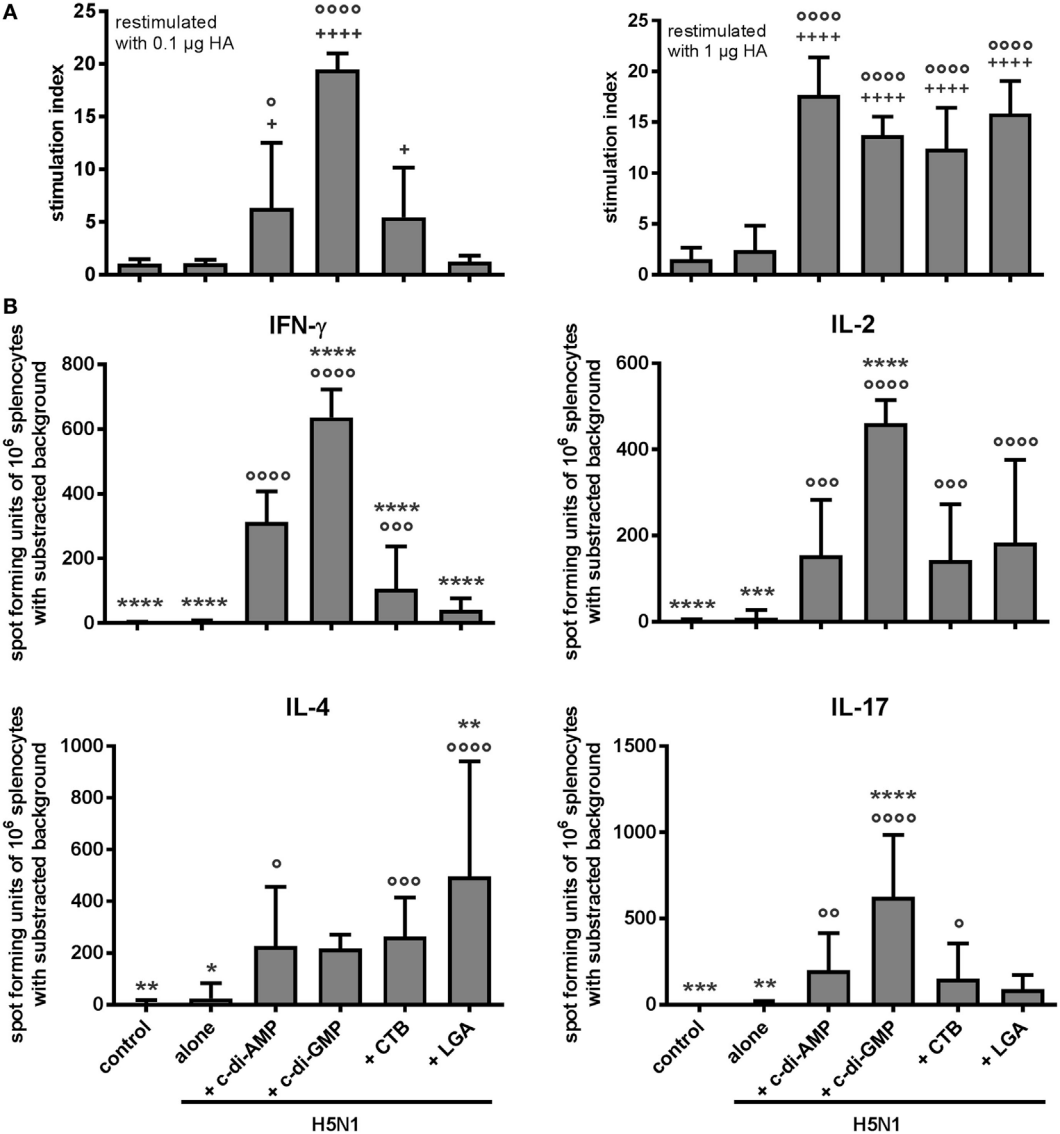
Cellular response. Groups of 3–5 BALB/c mice were immunized intranasally with PBS (control) or with two doses (21 days apart) of H5N1 virosomes (7.5 µg hemagglutinin), alone or adjuvanted with cyclic di-adenosine monophosphate (c-di-AMP) (5 µg), c-di-GMP (5 µg), cholera toxin subunit B (CTB) (10 µg), or last (or third) generation adjuvant (LGA) (7.5 µg). At 21 days after the second immunization, spleen cells were harvested, pooled, and restimulated with homologous H5N1 virosomes. **(A)** Proliferation. The proliferative response was measured by incorporation of ^3^H-thymidine (counts per minute, cpm). The stimulation index (cpm of restimulated sample vs. cpm of unstimulated sample) is shown from quadruplicates of *n* = 3 (PBS, alone), *n* = 2 (c-di-AMP, CTB), or *n* = 1 (c-di-GMP, LGA) independent experiments, as mean + SD. Statistically significant differences were measured by one-way ANOVA with Tukey’s *post hoc* test; differences from the negative control or antigen alone group are shown by plus signs or circles, respectively (^+^/°*p* < 0.05, ^++^/°°*p* < 0.01, ^+++^/°°°*p* < 0.001, ^++++^/°°°°*p* < 0.0001). **(B)** Cytokine production. The number of cytokine-producing cells was determined by ELISpot. Results are presented as spot-forming units of 10^6^ restimulated cells minus the background values from unstimulated cells. The mean + SD are shown from triplicates in two cell concentrations of *n* = 4 (PBS, alone), *n* = 3 (c-di-AMP, CTB), *n* = 2 (LGA), or *n* = 1 (c-di-GMP) independent experiments. Statistically significant differences were measured by one-way ANOVA with Tukey’s *post hoc* test; differences from the c-di-AMP or antigen alone group are shown by asterisks or circles, respectively (*/°*p* < 0.05, **/°°*p* < 0.01, ***/°°°*p* < 0.001, ****/°°°°*p* < 0.0001).

The current opinion on effectively combatting an influenza outbreak is that a vaccine should induce both humoral and cellular immune defenses (29). Accordingly, we analyzed the *ex vivo* proliferative capacity of spleen cells from the vaccinated animals. All tested adjuvants promoted vaccine-induced cellular immunity detectable by the proliferative capacity at relatively low doses of 1 µg/mL antigen (Figure 2A). The characteristic of the cell-mediated effector functions was elaborated in terms of the produced cytokines. Following antigen restimulation of the splenocytes, similar numbers of cells from the different adjuvanted groups were found to produce the Th2 cytokine IL-4; by contrast, both c-di-AMP and c-di-GMP groups gave significantly higher numbers of cells producing the Th1 cytokine IFN-γ when compared with the CTB or LGA group (Figure 2B).

This Th1/Th2 distribution was in line with the induced IgG subclasses and was also noted when measuring the cytokine concentrations in the supernatants from the restimulated spleen cells (Figure S1A in Supplementary Material). Further analysis of this cytokine profile induced by restimulation of the splenocytes confirmed the Th1 bias arising from the c-di-AMP-adjuvanted vaccination. High levels of both IFN-γ and TNF-α (Th1-associated cytokines) were observed, but less IL-4, IL-5, and IL-10 (Th2 cytokines) compared with restimulated splenocytes from the CTB- and LGA-adjuvanted groups. In addition, we found IL-17-producing cells in the c-di-AMP, c-di-GMP, and CTB groups, as well as high levels of IL-22 secretion with pro-inflammatory or regenerative potential; cells from the LGA group did not produce these cytokines upon restimulation, although a low level of IL-17-producing cells were observed (Figure 2; Figure S1A in Supplementary Material). Both, IL-17 and IL-22, are tissue-signaling cytokines that favor protection and regeneration of barrier organs, such as skin and lung (30).

**Figure 3:**
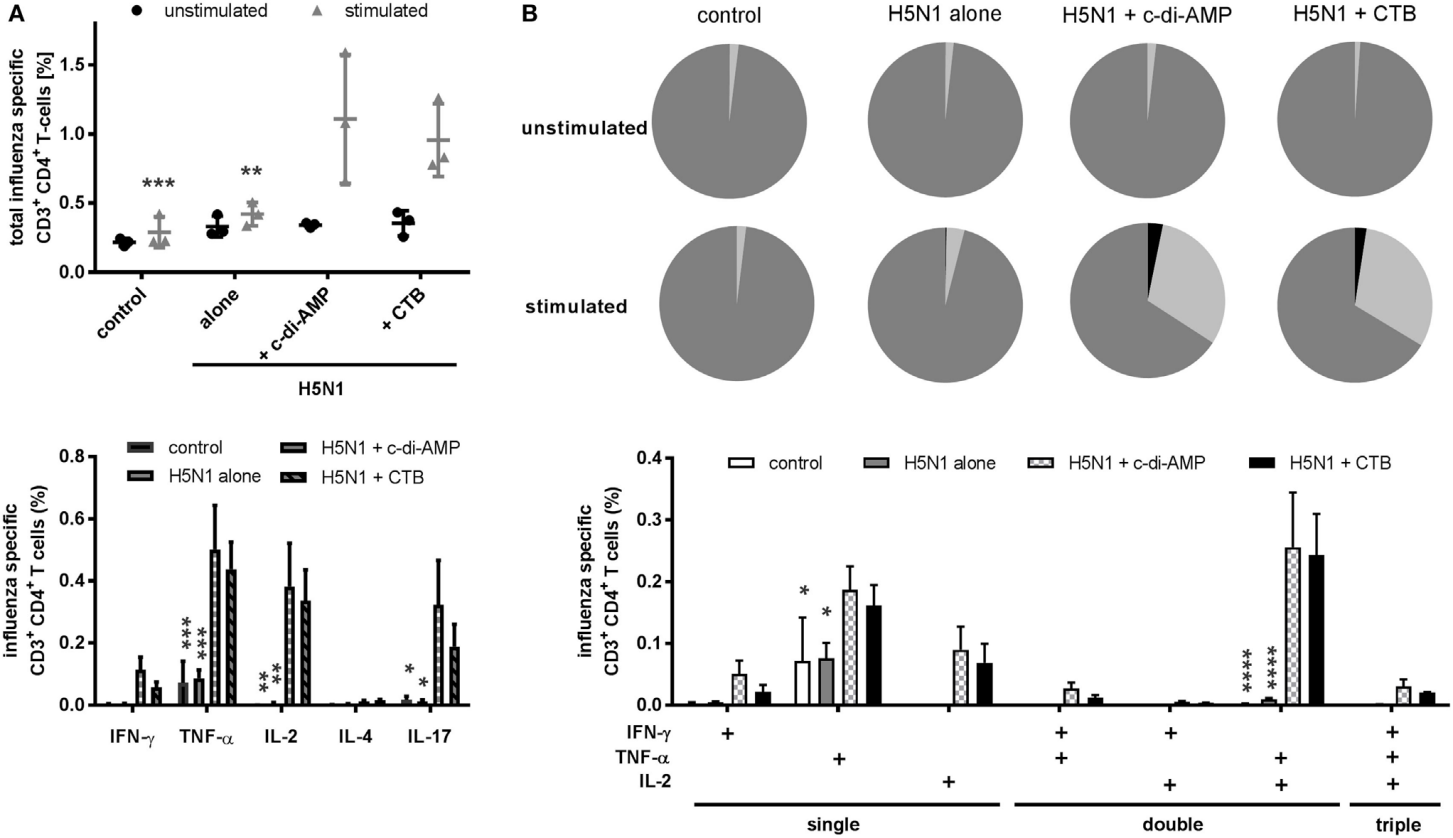
T cell quality. Groups of three BALB/c mice were immunized intranasally with PBS (control) or with two doses (21 days apart) of H5N1 virosomes (7.5 µg hemagglutination), alone or adjuvanted with cyclic di-adenosine monophosphate (c-di-AMP) (5 µg) or cholera toxin subunit B (CTB) (5 µg). At 21 days after the second immunization, spleen cells were harvested, restimulated with homologous H5N1 virosomes, intracellularly stained for Th cytokines (IFN-γ, IL-2, TNF-α, IL-4, and IL-17), and analyzed by flow cytometry. **(A)** CD4^+^ T cell producers. Upper panel: all CD4^+^ cells producing at least one of the measured cytokines were summed to quantify the frequency of influenza virus-specific Th cells. The mean ± SD is shown; each symbol represents one animal. Lower panel: the mean + SEM of all CD4^+^ cells producing at least the indicated cytokine is shown. The number of unstimulated cells was subtracted from the respective number of stimulated cells. **(B)** Multifunctional T cell activity. The pie charts show the proportion of single (dark gray), double (light gray), and triple (black) cytokine producers; the bar chart shows the frequency (mean + SEM) of single, double, and triple Th1 cytokine-producing CD4^+^ cells. The number of unstimulated cells was subtracted from the respective number of stimulated cells. Statistically significant differences were measured by two-way ANOVA with Tukey’s *post hoc* test; differences from the c-di-AMP group are shown by asterisks (*p < 0.05, **p < 0.01, ***p < 0.001, ****p < 0.0001).

The quality of the effector function was further investigated in terms of how the different adjuvants influenced induction of multifunctional T cells, which is reported to correlate with protection against influenza (31). The c-di-AMP effectively induced H5N1-specific CD3^+^CD4^+^ cells producing at least one of the measured cytokines, e.g., IL-2, IL-4, IL-17, IFN-γ, or TNF-α (Figure 3A, *upper panel* and Figure 6D). High frequencies of TNF-α positive cells were observed (Figure 3A, *lower panel*). Contrasting with the ELISpot and CBA data, cells from the c-di-AMP- and CTB-adjuvanted groups did not show a clear difference for TNF-α or IL-2 induction.

When attention turned to the multifunctional Th1 cells, characterized by the simultaneous production of two or more of the Th1 cytokines, such as IL-2, TNF-α, IFN-γ, IL-17, or IL-4, both c-di-AMP- and CTB-adjuvanted vaccines had induced high frequencies of double producers (>30%) compared to the controls (<5%) (Figure 3B, *upper panel pie charts*). A low level of triple producer cells were also detectable (around 3%), which were absent from the control groups (Figure 3B, *upper panel*). The antigen-specific cytokine production is shown in Figure 3B, *lower panel*. In both the c-di-AMP and CTB groups, CD4^+^ cells were identified to be producing a single Th1 cytokine (Figure 3B, *lower panel “single”*), or TNF-α in combination with IFN-γ, IL-2, or both (Figure 3B, *lower panel “double” and “triple,” respectively*). Double producers for IFN-γ and IL-2 were rare. As for the antigen alone group, antigen-specific cytokine induction was restricted to TNF-α single producer. The gating strategy of multifunctional T cells and an example how we analyzed the intracellular cytokine secretion of these CD3^+^CD4^+^ T cells is illustrated in Figures S4A,B in Supplementary Material.

### c-di-AMP Application Leads to Efficacious Induction of Protection against H5N1

**Figure 4:**
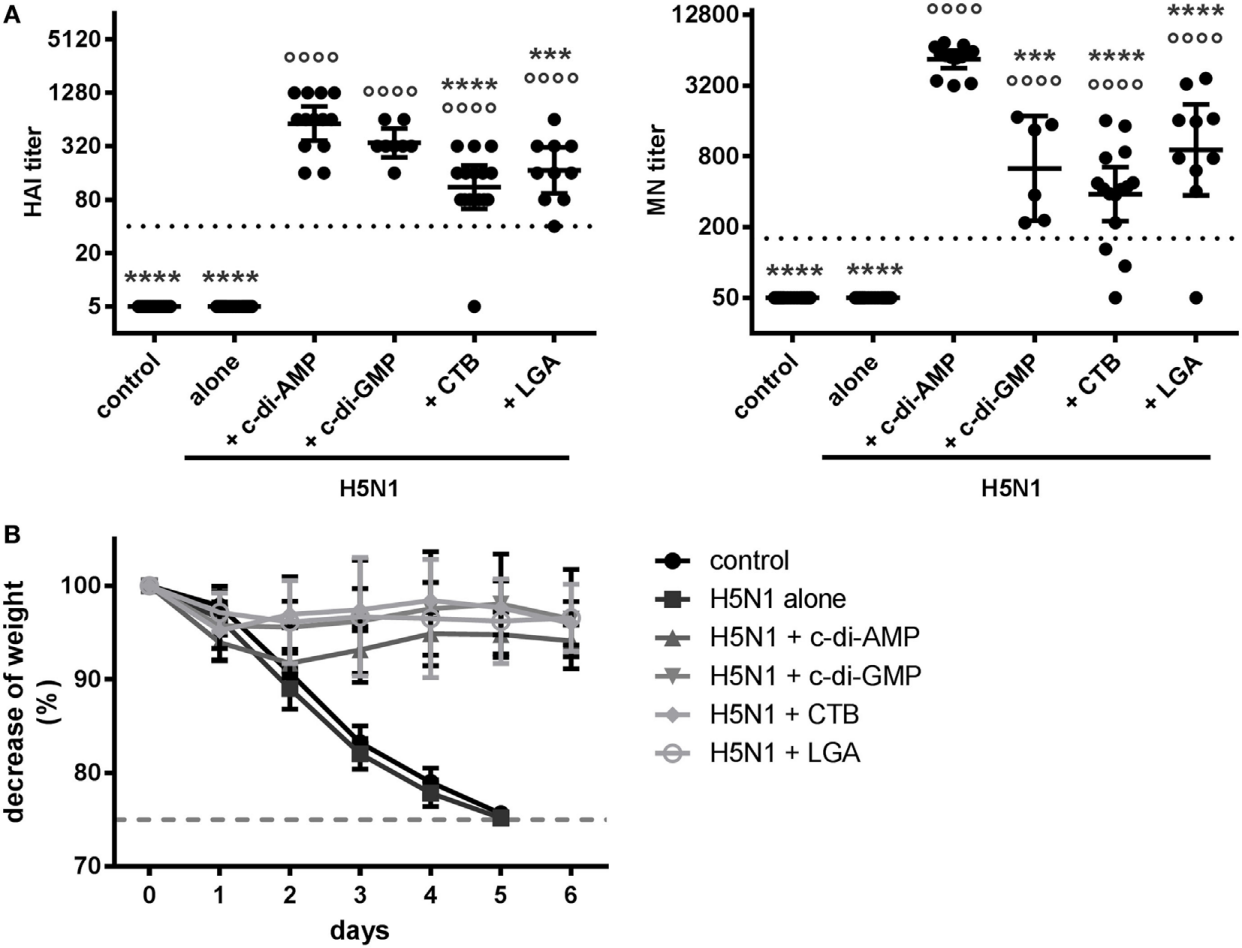
Protection. **(A)** Correlates of protection. Groups of 3–5 BALB/c mice were immunized intranasally with PBS (control) or with two doses (21 days apart) of H5N1 virosomes [7.5 µg hemagglutinin (HA)], alone or adjuvanted with cyclic di-adenosine monophosphate (c-di-AMP) (5 µg), c-di-GMP (5 µg), cholera toxin subunit B (CTB) (10 µg), or last (or third) generation adjuvant (LGA) (7.5 µg). At 21 days after the second immunization, blood was sampled and sera obtained. The HAI and MN titers against homologous virosomes were measured in *n* = 4 (alone), *n* = 3 (control, c-di-AMP, CTB), *n* = 2 (LGA), or *n* = 2 (c-di-GMP) independent experiments, presented as one symbol per animal. The lines represent the geometric mean of the titers with 95% CI. Negative samples were assigned a value below the detection limit (5 for HAI and 50 for MN) for calculation purposes. The dotted line represents the protective HAI titer of 40 or MN titer of 160, respectively. Statistically significant differences were measured by one-way ANOVA with Tukey’s *post hoc* test of the log_2_ normalized data; differences from the c-di-AMP or antigen alone group are shown by asterisks or circles, respectively (*/°*p* < 0.05, **/°°*p* < 0.01, ***/°°°*p* < 0.001, ****/°°°°*p* < 0.0001). **(B)** Protection against virus challenge infection. BALB/c mice were immunized intranasally with PBS (control, 11 animals) or with two doses (21 days apart) of H5N1 virosomes (7.5 µg HA), alone (9 animals) or adjuvanted with c-di-AMP (5 µg, 11 animals), c-di-GMP (5 µg, 9 animals), CTB (5 µg, 9 animals), or LGA (5 µg, 8 animals). Four weeks after the second immunization, mice were intranasally challenged with 2 × 10^3^ ffu of NIBRG-14; the mean percentage changes in body weight (± SD) were calculated. One control animal did not show any clinical symptoms or weight loss and was excluded. According to local regulations, animals showing more than 25% weight loss (dashed line) were euthanized.

In order to evaluate the functionality of the c-di-AMP-induced antibodies, we determined HAI and MN titers (Figure 4A), which are recognized as immunological correlates of protection against influenza viruses (32). Neither the negative control group nor the antigen alone group showed measurable HAI or MN titers. Adjuvant application clearly facilitated induction of HAI and MN titers, which should relate to protection (titers above 40 and 160, respectively). Interestingly, the c-di-AMP adjuvant group displayed significantly higher HAI and MN titers with a closer clustering of antibody titers than animals receiving vaccine formulated with c-di-GMP, CTB, or LGA. The consideration that the antibody titers were clearly reflecting correlates of protection prompted confirmation by assessing protection against challenge H5N1 infection (NIBRG-14 body weight over time post-challenge). Figure 4B shows that all mice vaccinated with adjuvanted H5N1 virosomes had only slight weight reduction during the first days after infection and recovered completely by day 6. In comparison, non-vaccinated mice or mice vaccinated with the virosomal vaccine alone showed unambiguous weight reduction, requiring euthanization when body weight loss reached max. 25%.

### Induction of Long-Lasting B Cells and Cross-Protection against Drifted H5N1 Strains

**Figure 5:**
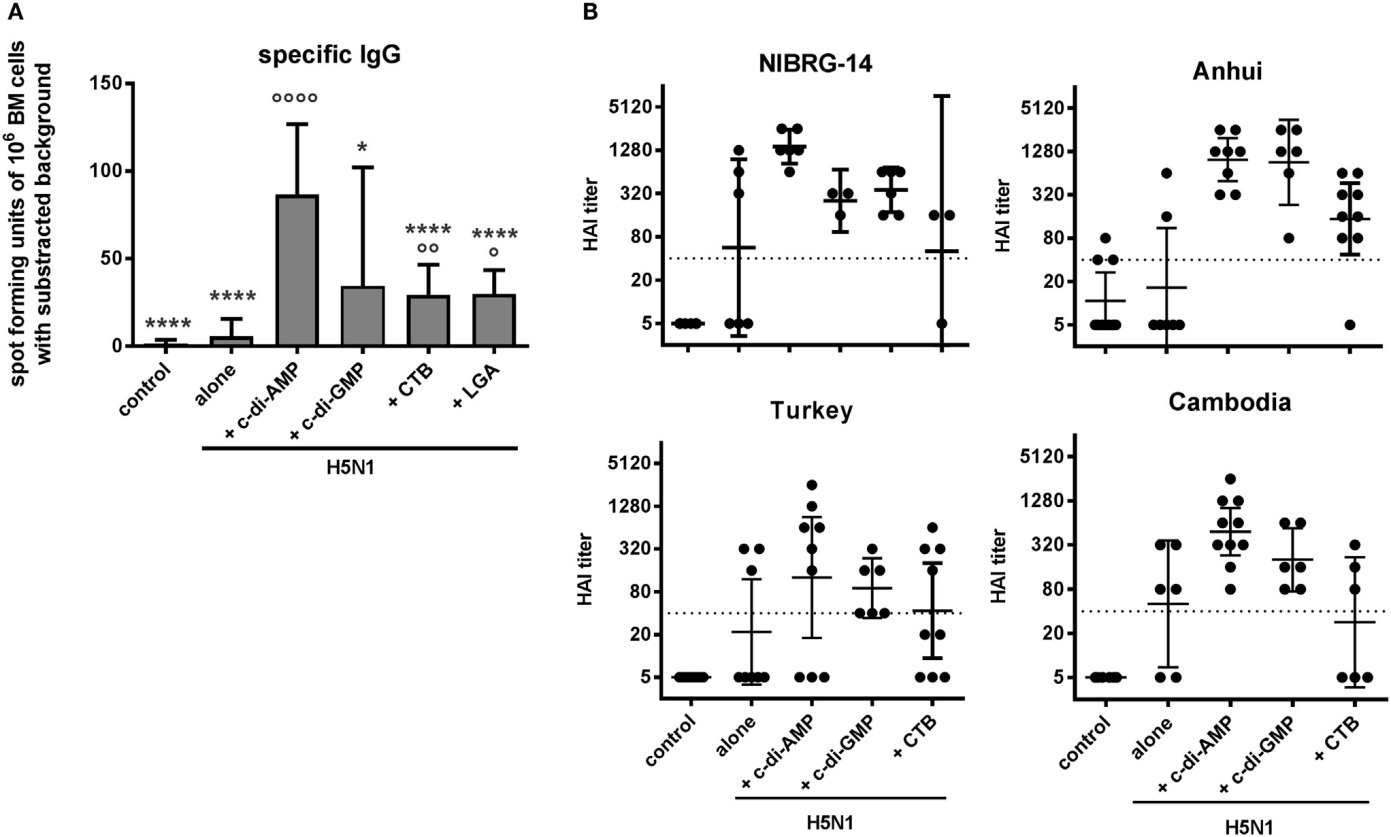
Memory and cross-reactivity. Groups of five BALB/c mice were immunized i.n. with PBS (control) or with two doses (21 days apart) of H5N1 virosomes (7.5 µg hemagglutinin HA), alone or adjuvanted with cyclic di-adenosine monophosphate (c-di-AMP) (5 µg), c-di-GMP (5 µg), cholera toxin subunit B (CTB) (10 µg), or last (or third) generation adjuvant (LGA) (7.5 µg). **(A)** Immunological memory. Two to three weeks after the second immunization, bone marrow (BM) cells were harvested, pooled, and restimulated with homologous H5N1 virosomes. The number of antigen-specific IgG-producing B cells derived from the BM was determined by ELISpot. Results are presented as antigen-specific spot-forming units of 10^6^ restimulated cells minus the background unstimulated values. The mean + SD is shown from quadruplicates in two cell concentrations of *n* = 3 (PBS, alone, CTB) or *n* = 2 (c-di-AMP, c-di-GMP, and LGA) independent experiments. Statistically significant differences were measured by one-way ANOVA with Tukey’s *post hoc* test; differences from the c-di-AMP or antigen alone group are shown by asterisks or circles, respectively (*/°*p* < 0.05, **/°°*p* < 0.01, ***/°°°*p* < 0.001, ****/°°°°*p* < 0.0001). **(B)** Cross-clade reactivity. Two to three weeks after the second immunization, blood was sampled and sera obtained. The HAI titers against the homologous vaccine strain and genetically distinct (clade) strains were measured and are presented as one symbol per animal. The lines represent the geometric mean of the titers with 95% CI; the dotted line shows the protective HAI titer of 40. Negative samples were assigned a value of 5 for calculation purposes. One experiment out of three independent experiments is shown.

Effective vaccination also requires memory cell induction to facilitate rapid responses upon re-encountering their specific antigen. In this context, the efficacy of the c-di-AMP adjuvanting was assessed in terms of vaccine-induced antigen-specific long-lasting B cells in the bone marrow (BM), measured by antigen-specific restimulation of IgG production by BM-derived B cells (Figure 5A). The c-di-AMP-adjuvanted group displayed high numbers of re-activated antigen-specific IgG-producing B cells. The number of cells was significantly higher than that obtained using BM cells from control animals.

A final test on the robustness of the induced immune response assessed the capacity of the H5N1 vaccine (NIBRG-14) to induce cross-clade antibody responses against drifted influenza virus strains. Figure 5B shows that control mice or mice vaccinated with H5N1 alone did not display evidence for cross-reactive responses. By contrast, mice vaccinated with H5N1 co-administered with c-di-AMP-adjuvanted vaccine did produce high HAI titers reactive against drifted virus strains, as exemplified with the A/Anhui/1/2005 (clade 2.3.4) virus shown in Figure 5B. Although the reactivities against the A/turkey/Turkey/1/2005 (clade 2.2.1) and A/Cambodia/R0405050/2007 (clade 1.1) viruses was less obvious, there was evidence of cross-reactive responses when the cyclic-di-nucleotides were employed as adjuvants, at least with individual animals, which was reduced or completely absent when CTB was co-administered.

### Influence of c-di-AMP Application on Dose Sparing

One important aspect of adjuvant application is the capacity to allow dose sparing. We, therefore, assessed how much the amount of the HA antigen (H5N1 virosome) could be reduced in combination with c-di-AMP. BALB/c mice were vaccinated by i.n. route with H5N1 virosomes adjuvanted with c-di-AMP at dosages ranging from 7.5 to 0.1 µg of HA, followed by analysis of humoral and cellular responses as well as protection from virus challenge. The humoral systemic response showed similar characteristics in all c-di-AMP-adjuvanted groups down to 0.5 µg HA (Table S1A in Supplementary Material). In this range, the IgG, IgG1, IgG2a, IgG2b, and IgG3 titers significantly increased compared to the antigen alone group, but with only little differences between the groups receiving reduced antigen doses. As for the group receiving only 0.1 µg HA adjuvanted with c-di-AMP, the induced antibody titers were clearly reduced, although still higher than those obtained with antigen alone, particularly for IgG2b. None of the groups showed an increase in the IgE titer.

**Figure 6:**
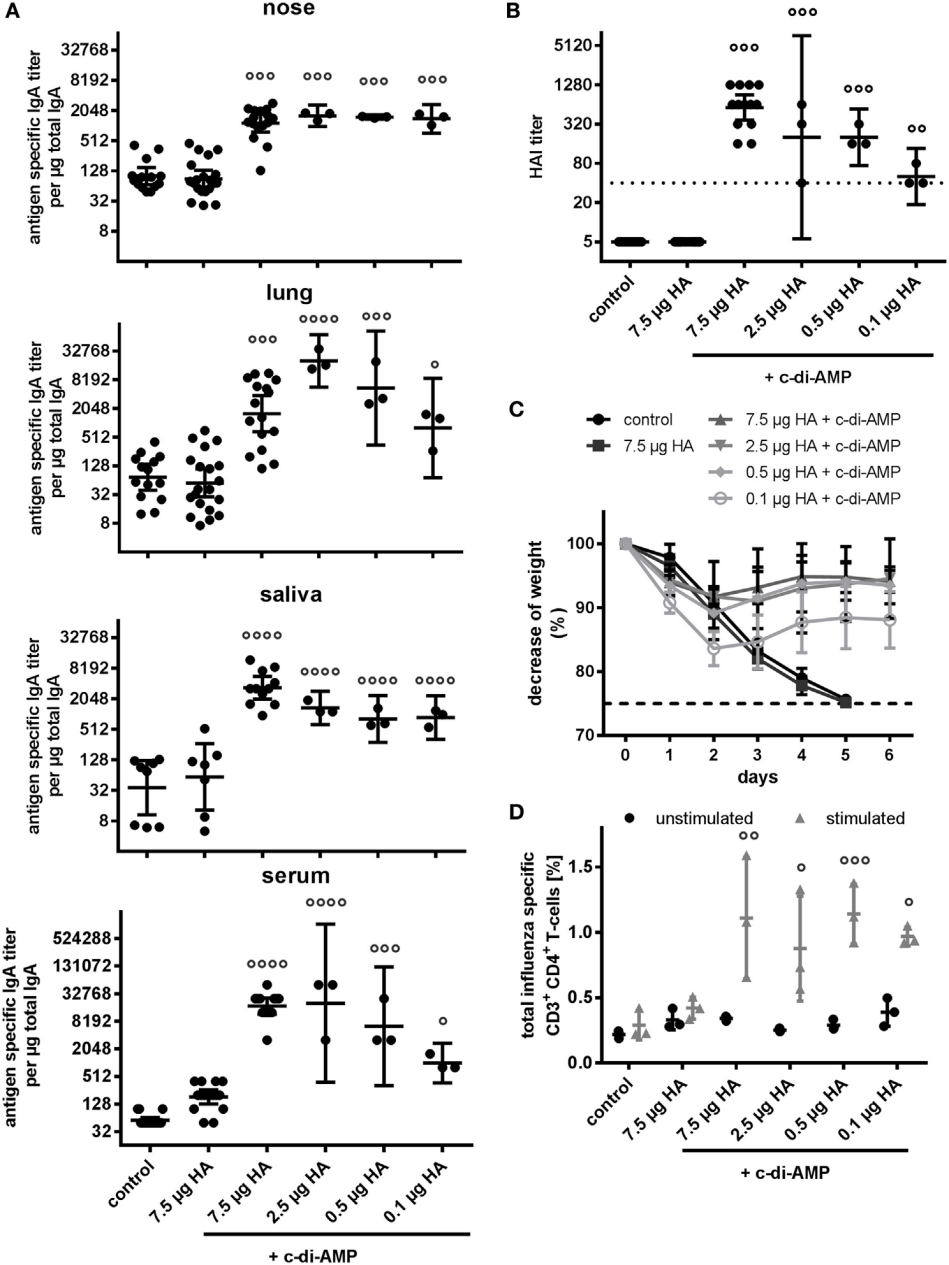
Dose sparing. Groups of three BALB/c mice were immunized intranasally with PBS (control) or with two doses (21 days apart) of H5N1 virosomes [7.5, 2.5, 0.5, or 0.1 µg hemagglutinin (HA)], alone (only 7.5 µg HA) or adjuvanted with cyclic di-adenosine monophosphate (c-di-AMP) (5 µg). **(A)** Mucosal antibody response. At 21 days after the second immunization, lung and nasal washes, as well as samples of saliva and serum were collected. Total and antigen-specific IgA was measured by ELISA. The geometric means with 95% CI are shown of the endpoint titer of antigen-specific IgA per μg total IgA in the case of the lavages, and the endpoint titer of antigen-specific IgA in the case of serum. Each symbol represents one animal. Statistically significant differences from the antigen alone group measured by one-way ANOVA with Dunnett’s *post hoc* test of log_2_ normalized data is shown by asterisks (*p < 0.05, **p < 0.01, ***p < 0.001, ****p < 0.0001). **(B)** Correlates of protection. At 21 days after the second immunization, blood was sampled and sera obtained. The hemagglutination inhibition (HAI) titers against homologous virosomes were measured and presented as one symbol per animal. The lines represent the geometric mean of the titers with 95% CI. Negative samples were assigned a value of 5 for calculation purposes. The dotted line represents the protective HAI titer of 40. Statistically significant differences from the antigen alone group measured by one-way ANOVA with Dunnett’s *post hoc* test of log_2_ normalized data is shown by circles (°*p* < 0.05, °°*p* < 0.01, °°°*p* < 0.001, °°°°*p* < 0.0001). **(C)** Virus Challenge. Four weeks after the second immunization, mice (PBS and 7.5 µg HA + c-di-AMP 11 animals, antigen alone and 2.5 µg HA, 0.5 µg HA, and 0.1 µg HA + c-di-AMP 9 animals) were intranasally challenged with 2 × 10^3^ ffu of NIBRG-14 and the mean percentage changes in body weight (± SD) were calculated. Weight data from one PBS animal that did not show any clinical symptoms was excluded. According to local regulations, animals showing more than 25% weight loss (dotted line) were euthanized. **(D)** T cell quality. At 21 days after the second immunization, spleen cells were harvested, restimulated with homologous H5N1 virosomes, intracellularly stained for Th cytokines (IFN-γ, IL-2, TNF-α, IL-4, and IL-17), and analyzed by flow cytometry. All CD4^+^ cells producing at least one of the measured cytokines were summed to quantify the frequency of influenza virus-specific Th cells. The mean ± SD is shown with each symbol representing one animal. Statistically significant differences from the antigen alone group measured by two-way ANOVA with Dunnett’s *post hoc* test are shown by circles (°*p* < 0.05, °°*p* < 0.01, °°°*p* < 0.001, °°°°*p* < 0.0001).

The same picture was observed when looking at serum IgA and mucosal antibody responses. As expected (Figure 1), adjuvanting with c-di-AMP significantly increased serum and mucosal antigen-specific IgA titers compared with antigen alone (Figure 6A). Moreover, this increase of titers in serum, lung, nose, and saliva was observed down to the lowest HA concentration used (Figure 6A). Only a slight HA-antigen dose dependency was observed with the induced titers in the serum and lung; as with the serum IgG, this was more apparent at the lowest antigen concentration of 0.1 µg HA. By contrast, the induced nasal and saliva IgA titers were similar to all the HA antigen concentrations tested, including the 0.1 µg HA.

When assessed in terms of the HAI correlates of protection, the c-di-AMP adjuvant also facilitated induction of protective HAI titers (>40), even at the lowest antigen concentrations (Figure 6B). However, the titer in the animals receiving the 0.1 µg HA antigen concentration was again the lowest, and greater confidence that the titers would correlate to protection was obtained with the other groups, which still reflected a dose-sparing capacity for the c-di-AMP adjuvant.

This dose sparing, which is observed when monitoring the humoral response, was reflected by a similar influence on the cellular response (Figure S1 in Supplementary Material). Using the reduced HA antigen doses down to 0.5 µg, application of c-di-AMP also facilitated induction of cellular responses, measured in terms of high antigen-specific proliferation upon restimulation; a reduced proliferative response was observed with cells from animals receiving only 0.1 µg HA (Figure S1A in Supplementary Material). A similar dose-dependent response was observed in terms of cells positive for Th1 cytokines (IL-2 and IFN-γ) and the Th17 cytokine IL-17, in that cells from the c-di-AMP-adjuvanted groups receiving 7.5–0.5 µg of HA had similar high responses (Figure S1B in Supplementary Material). Although cells positive for the Th2 cytokine IL-4 were fewer than for the Th1 and Th17 cytokines, there were still similar levels detected in the groups receiving 7.5–0.5 µg of HA.

Further confirmation of the dose sparing permitting cellular responses was observed when measuring different cytokines in the supernatant of *in vitro*-restimulated spleen cells (Figures S1B and S3B in Supplementary Material) and by FACS analysis for multifunctional T cells (Figure 6D and Figure S1C in Supplementary Material). In all c-di-AMP groups, induction of H5N1-specific CD3^+^CD4^+^ cells led to production of at least one of the intracellular cytokines measured, such as IL-2, IL-4, IL-17, IFN-γ, and/or TNF-α. High frequencies of cells producing single cytokines (IL-2, TNF-α, and IL-17) were noted (Figures S1C,D in Supplementary Material). In contrast to the ELISpot data (Figure S1B in Supplementary Material), only low frequencies of IFN-γ- and IL-4-producing T cells were detected. Importantly, in all animals receiving H5N1 plus c-di-AMP, an increase in double producer and triple producer cells (e.g., IL-2, TNF-α, and IFN-γ) was observed (Figure S1D in Supplementary Material). The antigen-specific cytokine production of stimulated vs. unstimulated samples showed that the domination by Th1 cells producing TNF-α alone or in combination with IL-2 was maintained even with the reduced dosage of HA in the vaccine (Figure S1D in Supplementary Material). Reflecting the dose-sparing capacity offered by the c-di-AMP adjuvant in terms of induced humoral and cell-mediated responses, as well as the protection against the H5N1 virus (NIBRG-14) challenge infection. All animals receiving 7.5–0.5 µg HA in combination with c-di-AMP showed only slight weight reduction and recovered completely by day 6 (Figure 6C). Animals receiving only 0.1 µg HA in combination with c-di-AMP showed a more marked weight loss, but this was still less than that observed with antigen alone; in contrast to the latter group, animals receiving the 0.1 µg HA in combination with c-di-AMP recovered. Also reflecting the observations on the immune responses, there was no significant difference in the animal groups receiving the 7.5–0.5 µg HA doses.

### Influence of c-di-AMP Application by the Sublingual Route

The potential for s.l. vaccination as a potential alternative mucosal route to overcome some of the drawbacks of i.n. delivery was also assessed with BALB/c mice vaccinated using H5N1 virosomes alone or adjuvanted with c-di-AMP (33). The humoral systemic response was comparable to that observed in i.n. vaccinated mice (Table S1B in Supplementary Material). Antigen-specific IgG, IgG1, IgG2a, IgG2b, and IgG3 titers were significantly increased in animals receiving H5N1 in combination with c-di-AMP, c-di-GMP, or CTB, as compared to the antigen alone group. By contrast, the LGA was not able to improve the humoral response *via* the s.l. route. Looking at the levels of the IgG subtypes, a more balanced Th1/Th2 ratio was observed in the c-di-AMP group, compared with a more Th2-biased ratio in the CTB group.

**Figure 7:**
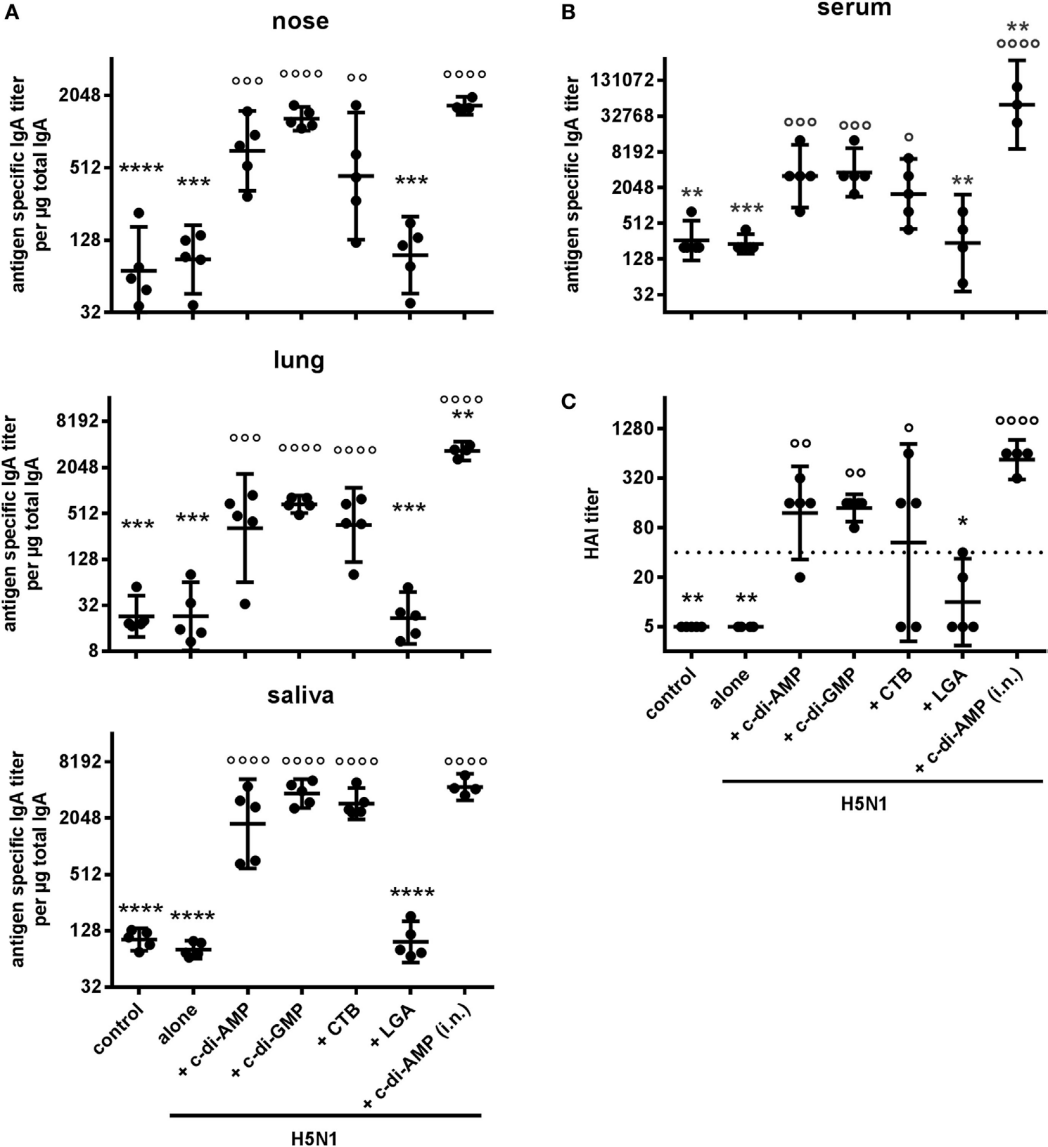
Alternative mucosal routes. Groups of five BALB/c mice were immunized sublingually with PBS (control) or with two doses (21 days apart) of H5N1 virosomes [2 µg hemagglutinin (HA)], alone or adjuvanted with cyclic di-adenosine monophosphate (c-di-AMP) (5 µg), c-di-GMP (5 µg), cholera toxin subunit B (CTB) (10 µg), or last (or third) generation adjuvant (LGA) (7.5 µg). A control group received H5N1 virosomes (7.5 µg HA) + c-di-AMP (5 µg) by i.n. route. **(A)** Mucosal responses in local tissues. **(B)** IgA antibody response in sera. At 21 days after the second immunization, the lung and nasal washes, as well as the saliva and serum samplings were obtained. Total and antigen-specific IgA was measured by ELISA. The geometric mean with 95% CI is shown for the endpoint titer of antigen-specific IgA per μg total IgA in the case of lavages, and the endpoint titer of antigen-specific IgA in the case of serum. Each symbol represents one animal. **(C)** Correlates of protection. At 21 days after the second immunization, blood was sampled and sera obtained. The hemagglutination inhibition (HAI) titers against homologous virosomes were measured and presented as one symbol per animal. The lines represent the geometric mean of the titers with 95% CI. Negative samples were assigned a value of 5 for calculation purposes. The dotted line represents the protective HAI titer of 40. Statistically significant differences were measured by one-way ANOVA with Tukey’s *post hoc* test of log_2_ normalized data; differences from the c-di-AMP s.l. or the antigen alone group are shown by asterisks or circles, respectively (*/°*p* < 0.05, **/°°*p* < 0.01, ***/°°°*p* < 0.001, ****/°°°°*p* < 0.0001).

The local IgA mucosal responses (Figure 7A) and the systemic IgA response (Figure 7B) were also enhanced in animals receiving H5N1 virosomes in combination with c-di-AMP, c-di-GMP, or CTB *via* the s.l. route. These results were again comparable to those obtained from the vaccinations *via* the i.n. route. Furthermore, protective HAI titers were observed in animals receiving c-di-AMP or c-di-GMP as adjuvant, although slightly reduced compared with the i.n. route (Figure 7C). While the animals receiving vaccines adjuvanted with the cyclic d-nucleotides showed reasonable clustering of the titers from individual animals, there was considerable variation with animals receiving CTB-adjuvanted vaccine. Indeed, some of the latter group did not produce HAI titers reaching the protective levels. Moreover, none of the animals receiving LGA-adjuvanted vaccine produced titers to the minimum protective level, this lack of response confirming the LGA adjuvant to be unsuitable for the s.l. route of vaccination.

The proliferative responses induced by restimulation assays with splenocytes from animals receiving vaccines adjuvanted with c-di-AMP, c-di-GMP, or CTB by s.l. administration reflected those obtained with cells from mice vaccinated by the i.n. route (data not shown). Cells were also positive for IL-4, IL-2, and IL-17 in these groups and, in line with the observed Th responses, high levels of IFN-γ positive cells were found with cells from the groups receiving cyclic di-nucleotides (Figure S3A in Supplementary Material). The induced cytokine profile was confirmed by FlowCytomix (data not shown) and similar results were observed by FACS when checking for multifunctional T cells. Animals receiving c-di-AMP or CTB as adjuvant developed H5N1-specific CD3^+^CD4^+^ cells producing IL-2, IL-4, IL-17, IFN-γ, or TNF-α (Figures S2B,C in Supplementary Material). In addition, only a few cells producing IL-4 could be detected. With cells from the c-di-AMP group, the detectability of IFN-γ-producing T cells was in line with the ELISpot data. Nevertheless, the i.n. route remained more effective at inducing antigen-specific T cells.

As for the multifunctional Th1 cells, cells were detected capable of producing two or more of the Th1 cytokines IL-2, TNF-α, and IFN-γ (Figure S2C in Supplementary Material). While multifunctional cells could not be detected in the CTB group, low frequencies of double (e.g. TNFα^+^IL-2^+^) and triple (TNFα^+^IL-2^+^IFNγ^+^) producer cells were isolated from the group vaccinated with c-di-AMP as adjuvant. Within the double producer population, cells expressing TNF-α in combination with IL-2 dominated, followed by those expressing TNF-α in combination with IFN-γ. These characteristics are similar to those obtained when the vaccines were administered i.n, although the frequencies of cytokine-producing cells were lower (Figure S3A in Supplementary Material).

## Discussion

Epidemic and pandemic outbreaks caused by emerging influenza virus strains, such as the H1N1 of 2009 and the novel avian strains H5N5, H5N8, or H7N9, together with a growing number of high-risk individuals such as elderly or immunosuppressed patients, highlight the need for more effective influenza vaccines. The majority of licensed seasonal vaccines induce humoral responses which correlate with an effective immunity in healthy young adults. However, in susceptible groups such as the elderly, or in the case of avian influenza, humoral immunity appears to be insufficient for conferring efficacious immunity providing protection against severe disease. It is also important that vaccines efficiently stimulate influenza virus-specific Th and cytotoxic T cells to combat infection (29). In these contexts, adjuvants offer potential both for improved vaccine efficacy and modulating the immune defense compartments induced by the vaccination. In addition, adjuvants can facilitate dose sparing, particularly useful during a pandemic. Considering the highly contagious and communicable nature of influenza, efficacious prophylaxis would also benefit from an effective mucosal vaccination strategy to block infection at a very early stage and reduce the risk of horizontal transmission (34). Accordingly, this study explored the potential of c-di-AMP as a mucosal adjuvant, applied with a virosome-based vaccine against influenza virus H5N1.

Both humoral and cellular immune responses against influenza virus were evaluated after i.n. or s.l. vaccination with H5N1 virosomes alone or in combination with c-di-AMP or c-di-GMP. As gold standard formulations, virosomes were co-administered with LGA or CTB. In line with previous studies, c-di-AMP vaccinated animals showed balanced production of IgG1 and IgG2a subclass titers, which correlate to a balanced Th1/Th2 response; by contrast, CTB- and LGA-induced dominant IgG1 responses (17, 18). The humoral immune response profiles were confirmed by the cellular response and respective cytokine profiles. While cyclic di-nucleotides, such as c-di-AMP or c-di-GMP, facilitated a balanced Th1/Th2/Th17 response, animals vaccinated with formulations using CTB or LGA showed a Th2-biased profile. Therefore, despite the efficacious induction of responses by vaccines formulated with CTB and LGA, the c-di-AMP formulation proved to be a more appropriate adjuvant for an influenza vaccine considering the overall induced immune response (28). Th1 cells are important for the elimination of an intracellular pathogen such as influenza virus, whereas Th2 cells are critical for effective humoral immunity, which facilitates affinity maturation and class switch essential for virus neutralization (35, 36). Furthermore, induction of IL-10, which promotes IgA switch and displays broad anti-inflammatory properties (37), is also involved in self-regulation of Th1 responses (38). This aspect is particularly important for the safety profile of new adjuvants, because it can reduce the likelihood of adverse side effects by limiting immune reactions and diminishing the risk of immuno-pathological developments (Figures S3A,B in Supplementary Material).

Interestingly, Th17 cell induction was also influenced by c-di-AMP, c-di-GMP, and CTB, but not by LGA. The role of Th17 in influenza virus infection is controversial, ranging from studies reporting beneficial to detrimental effects (39, 40), although this variation in the consequence of Th17 induction may be reflecting the combination with other cytokine activities. Certainly, Th17 is important for host response at mucosal sites (41, 42) and plays a key role in the induction of IgA, including upregulation of the Ig receptor and transport of secretory IgA (43). The observed high levels of serum IgA indicate a strong activation of this class switching and may reflect mucosal B lymphocyte induction; indeed, high local IgA titers were obtained. In this context, adjuvanting with c-di-AMP showed superiority over CTB, a gold standard for mucosal adjuvants (44). In addition, the use of the c-di-AMP as adjuvant proved superior to CTB and LGA in terms of promoting the induction of protective HAI and MN titers. This may relate to the observed differential IgG subclass induction patterns, because each subclass exhibits different biological activities (45). Nevertheless, all the tested adjuvants proved efficient in facilitating vaccine-induced protection of the mice against infection.

Cases of Bell’s palsy have been reported in association with vaccines adjuvanted with LT and enzymatically inactive derivatives (9, 46). These adjuvants bind to receptors in the axons from the olfactory nerves, which promotes retrograde homing to the central nervous system (46). Thus, despite the fact that all described cases were related to the same class of adjuvants, the adverse event tainted the general image of i.n. vaccination applicability. The consequent drop in confidence from scientists, vaccinologists, regulators, and the public rendered more difficult the licensing of i.n. vaccines containing new adjuvants. Therefore, alternative mucosal administration routes have come increasingly into focus. In this context, we administered our experimental vaccine adjuvanted with c-di-AMP *via* the promising s.l. route. Both humoral and cellular immune responses were obtained, comparable in quality to those observed after i.n. vaccination. The s.l. and i.n. vaccination with c-di-AMP-adjuvanted virosomes elicited responses which were similar in quality but reduced in magnitude for the s.l. route, relating to observations employing the c-di-GMP adjuvant (20). Interestingly LGA, which promoted clear responses following i.n. vaccination, showed poor activity when administered by the s.l. route. The s.l. vaccination also promoted similar high HAI titers comparable to those observed following i.n. immunization. No IgE induction was observed using c-di-AMP as adjuvant regardless of the administration route (i.n. and s.l.), which is also in line with previous findings (18).

A number of recent studies have described the importance of multifunctional Th cells, and it is reported that these cells are associated with efficient protection against infection (31, 47). Furthermore, a correlation between multifunctional cells and strong antibody responses after vaccination against influenza has been suggested (21). Application of c-di-AMP as adjuvant did facilitate vaccine induction of double and triple producers, dominated by cells double positive for IL-2 and TNF-α, which is in agreement with studies using the c-di-GMP adjuvant (19–21). By contrast, Darrah et al. and Forbes et al. reported responses dominated by TNF-α/IFN-γ double positive cells (31, 47), but they employed different antigens, which might in part explain the observed differences. Interestingly, the TNF-α/IL-2 phenotype has been suggested to correlate with a good T cell memory (48), supporting our finding of high numbers of long-lasting B cells in BM secreting antigen-dependent antibodies which were correlated to protection (49). Comparing different mucosal routes showed that i.n. application was more effective at inducing multifunctional T cells. However, higher frequencies of double and triple cytokine producers were obtained from the c-di-AMP-vaccinated animals, which was not the case when using the gold standard CTB as adjuvant.

As new influenza virus variants emerge due to frequent antigen changes resulting from point-mutations (drift) or gene segment rearrangements (shift), a cross-protective capacity of vaccines is highly desirable. Testing the ability of our chosen vaccine formulation to induce cross-protection against drifted H5N1 strains, we observed high HAI titers; it is considered that cross-clade protection will be even more striking *in vivo*. In this regard, c-di-AMP-dependent vaccine induction of IgA could play a key role due to the importance of IgA promoting cross-protection *via* immune exclusion, broadening immune memory and balancing pro-inflammatory responses (50, 51).

The final component of the present studies demonstrated clear dose-sparing capacities when c-di-AMP was employed for vaccine formulations. Comparable HA-specific immune responses were obtained at HA antigen doses ranging from 7.5 down to 0.5 µg. Even with an antigen concentration of 0.1 µg, both protective levels of HAI titers and an ultimate protection of mice against influenza virus H5N1 challenge infection were observed. This protection with the 0.1 µg HA antigen can only be regarded as partial due to the weight reduction observed after challenge, which correlated with diminished HAI and MN titers and overall reduced cellular and humoral responses compared to the higher doses. Nevertheless, application of c-di-AMP as adjuvant did permit dose sparing from 7.5 down to 0.5 µg, with even the 0.5 µg inducing effective HAI titers and protecting the mice against challenge infection to the same degree as the higher 7.5 µg dose. Svindland et al. also described dose-sparing capacities for the c-di-GMP adjuvant, but they observed diminished humoral responses already at 1.5 µg antigen. It remains to be elucidated whether this difference is due to the use of different vaccine antigens (such as inactivated influenza subunit vaccine NIBRG-14) or the specific adjuvant (19).

Overall, our results demonstrate that c-di-AMP contributes to the generation of a microenvironment that is conducive to the stimulation of both humoral and cellular immune responses and this is obtainable at systemic and mucosal levels. These induced immune defenses protect the vaccinated individuals against viral challenge, also conferring protection against drifted H5N1 strains, and the formulation allows effective dose sparing. Thus, c-di-AMP represents a promising adjuvant for developing mucosal vaccines against this important human pathogen and facilitates application of the vaccine by the alternative sublingual mucosal vaccination route, which is currently showing particular promise.

## Ethics Statement

All animal experiments were approved by the institutional ethical board and conducted in accordance to the regulations of the local government of Lower Saxony (Germany; No. 509.42502 04 017.08).

## Author Contributions

Conception/design of study: TE, JD, PB, SW, KS, GP, and CG. Acquisition of data: TE, JD, PB, SW, and KS. Analysis/interpretation of data: TE, JD, KS, RC, KM, and CG. Drafting the manuscript: TE, JD, KS, CG, RC, and KM. Important intellectual content: TE, JD, PB, SW, KS, GP, CG, RC, and KM. Approval of manuscript: TE, JD, PB, SW, KS, GP, CG, RC, and KM.

## Conflict of Interest Statement

CG and TE are named as inventors in a patent application covering the use of c-di-AMP as adjuvant (PCT/EP 2006010693). This does not alter our adherence to the Frontier in Immunology policies on sharing data. The other authors declared no conflict of interest.
