# Supplementary material for: Mucosal Administration of Cycle-Di-Nucleotide-Adjuvanted Virosomes Efficiently Induces Protection against Influenza H5N1 in Mice
Source: Front Immunol. 2017 Sep 28;8:1223. doi: 10.3389/fimmu.2017.01223 (PMC5624999; doi:10.3389/fimmu.2017.01223)

**Supplement material:**

# **Table S1: Systemic antibody response.**

Serum antigen specific IgG, subclasses (IgG1, IgG2a, IgG2b and IgG3) and total IgE were measured 21 days after the second immunization by ELISA. The geometric mean of the endpoint titer is presented with the 95% CI in parentheses. **(A) Dose sparing.** Groups of 3 BALB/c mice were immunized intranasally either with PBS (control) or with two doses (21 days apart) of H5N1 virosomes (7.5 µg, 2.5 µg, 0.5 µg or 0.1 µg HA) alone (only 7.5 µg HA) or adjuvanted with c-di-AMP (5 µg). Statistically significant differences to the antigen alone group measured by one-way ANOVA with Dunnett’s post hoc test of the log2 normalized data are shown by circles (° p<0.05, °° p<0.01, °°° p<0.001, °°°° p<0.0001). **(B) Alternative mucosal routes.** Groups of 5 BALB/c mice were immunized sublingually either with PBS (control) or with two doses (21 days apart) of H5N1 virosomes (2.2 µg HA) alone or adjuvanted with c-di-AMP (5 µg), c-di-GMP (5 µg), CTB (10 µg) or LGA (7.5 µg). Statistically significant differences as measured by one-way ANOVA with Tukey’s post hoc test of the log2 normalized data and differences to the c-di-AMP group are shown by asterisks (* p<0.05, ** p<0.01, *** p<0.001, **** p<0.0001).

| A | **Control** | **H5N1 alone**  **(7.5 µg)** | **H5N1 (7.5 µg) + c-di-AMP** | **H5N1 (2.5 µg) + c-di-AMP** | **H5N1 (0.5 µg)+ c-di-AMP** | **H5N1 (0.1 µg) + c-di-AMP** |  |
| --- | --- | --- | --- | --- | --- | --- | --- |
| **IgG** | 6.30E+02  (2.33E+02, 1.70E+03) | 1.59E+03  (2.17E+02, 1.16E+04) | 8.13E+05°°°°  (3.01E+05, 2.20E+06) | 4.06E+05°°°°  (2.93E+04, 5.64E+06) | 5.12E+05°°°°  (9.15E+04, 2.87E+06) | 1.01E+04  (7.26E+02, 1.40E+5) |  |
| **IgG1** | 5.00E+02°  (5.00E+02, 5.00E+02) | 1.27E+04  (6.60E+02, 2.45E+06) | 1.02E+06°°  (1.02E+06, 1.02E+06) | 3.23E+05°  (8.95E+03, 1.16E+07) | 3.23E+05°  (4.42E+04, 2.36E+06) | 2.02E+04  (1.45E+03, 2.80E+05) |  |
| **IgG2a** | 5.00E+02°  (5.00E+02, 5.00E+02) | 1.00E+03  (1.79E+02, 5.60E+03) | 3.23E+05°°°  (4.42E+04, 2.36E+06) | 4.06E+05°°°°  (1.60E+03, 1.03E+08) | 3.23E+05°°°  (1.19E+05, 8.72E+05) | 4.00E+03  (7.15E+02, 2.24E+04) |  |
| **IgG2b** | 7.94E+02  (2.94E+02, 2.15E+03) | 6.30E+01  (2.33E+01, 1.70E+03) | 5.12E+05°°°°  (9.15E+04, 2.87E+06) | 3.23E+05°°°°  (2.33E+04, 4.48E+06) | 2.56E+05°°°°  (2.56E+05, 2.56E+05) | 1.27E+04°°  (9.15E+02, 1.76E+05) |  |
| **IgG3** | 5.00E+02  (5.00E+02, 5.00E+02) | 6.30E+02  (2.33E+02, 1.70E+03) | 4.03E+04°°°  (1.12E+03, 1.45E+06) | 4.03E+04°°°  (1.12E+03, 1.45E+06) | 1.27E+04°°  (4.70E+03, 3.43E+04) | 2.52E+03  (3.45E+02, 1.84E+04) |  |
| **IgE** | 1.00E+02  (1.00E+02, 1.00E+02) | 1.26E+02  (4.66E+01, 3.41E+02) | 1.00E+02  (1.00E+02, 1.00E+02) | 2.00E+02  (1.01E+01, 3.95E+03) | 1.00E+02  (1.00E+02, 1.00E+02) | 2.52E+02  (9.33E+01, 6.81E+02) |  |
|  |  |  |  |  |  |  |  |
|  |  |  |  |  |  |  |  |
| B | **Control** | **H5N1 alone**  **(7.5 µg)** | **H5N1**  **+ c-di-AMP** | **H5N1**  **+ c-di-GMP** | **H5N1**  **+ CTB** | **H5N1**  **+ LGA** | **H5N1 (i.n.)**  **+ c-di-AMP** |
| **IgG** | 5.74E+02****  (3.90E+02, 8.44E+02) | 1.60E+04****  (3.20E+03, 8.01E+04) | 1.02E+06  (3.57E+05, 2.94E+06) | 2.35E+06  (1.15E+06, 4.83E+06) | 6.76E+05  (1.13E+06, 4.03E+06) | 2.11E+04***  (2.24E+03, 1.99E+05) | 2.05E+06  (5.73E+05, 7.32E+06) |
| **IgG1** | 5.00E+02****  (5.00E+02, 5.00E+02) | 2.43E+04**  (3.34E+03, 1.76E+05) | 8.91E+05  (4.34E+05, 1.83E+06) | 1.02E+06  (5.57E+05, 1.88E+06) | 7.76E+05  (1.45E+05, 4.15E+06) | 9.19E+03***  (4.28E+02, 1.97E+05) | 1.22E+06  (4.24E+05, 3.50E+06) |
| **IgG2a** | 5.00E+02****  (5.00E+02, 5.00E+02) | 6.96E+03****  (1.08E+03, 4.50E+04) | 6.76E+05  (3.13E+05, 1.46E+06) | 2.35E+06  (1.15E+06, 4.83E+06) | 2.94E+05  (4.55E+04, 1.90E+06) | 1.21E+04***  (1.28E+03, 1.14E+05) | 1.02E+06  (2.15E+05, 4.87E+06) |
| **IgG2b** | 5.00E+02****  (5.00E+02, 5.00E+02) | 6.06E+03****  (9.20E+02, 4.00E+04) | 6.76E+05  (4.22E+05, 1.08E+06) | 1.55E+06  (5.82E+05, 4.14E+06) | 5.88E+05  (2.86E+05, 1.21E+06) | 6.06E+03****  (4.72E+02, 7.79E+04) | 5.12E+05  (2.08E+05, 1.26E+06) |
| **IgG3** | 5.00E+02***  (5.00E+02, 5.00E+02) | 6.60E+02**  (4.12E+02, 1.06E+03) | 1.06E+04  (3.96E+03, 2.82E+04) | 1.84E+04  (5.98E+03, 5.65E+04) | 6.06E+03  (5.93E+02, 6.20E+04) | 1.74E+03  (3.73E+02, 8.12E+03) | 1.08E+05*  (6.20E+04, 1.87E+05) |
| **IgE** | 2.64E+02  (1.65E+02, 4.23E+02) | 5.28E+02  (3.29E+02, 8.46E+02) | 4.00E+02  (2.17E+02, 7.35E+02) | 4.00E+02  (1.18E+02, 1.35E+03) | 3.48E+02  (1.70E+02, 7.15E+02) | 6.96E+02  (2.71E+02, 1.79E+03) | 2.38E+02  (1.37E+02, 4.13E+02) |
|  |  |  |  |  |  |  |  |

# **Figure S1: Dose sparing, cellular response.**

Groups of 3 BALB/c mice were immunized intranasally either with PBS (control) or with two doses (21 days apart) of H5N1 virosomes (7.5 µg, 2.5 µg, 0.5 µg or 0.1 µg HA), alone (only 7.5 µg HA) or adjuvanted with c-di-AMP (5 µg). At 21 days after the second immunization, spleen cells were harvested, pooled and restimulated with homologous H5N1 virosomes. **(A) Proliferation.** The proliferative response was measured by incorporation of thymidine (counts per minute, cpm). The stimulation indices (cpm of restimulated sample vs. cpm of unstimulated sample) from quadruplicates are shown as the mean + SD. **(B) Cytokine production.** The number of cytokine-producing cells was determined by ELISpot. Results are presented as spot forming units of 10^6^ restimulated cells minus the values obtained with unstimulated cells. The mean ± SD from triplicates in two cell concentrations is shown. **T cell quality.** At 21 days after the second immunization, spleen cells were harvested, restimulated with homologous H5N1 virosomes, intracellularly stained for the Th cytokines IFN-γ, IL-2, TNF-α, IL-4 and IL-17 and analyzed by flow cytometry. **(C)** The frequency of CD4^+^ cells producing at least the indicated cytokine is shown as mean + SEM; the number of unstimulated cells was subtracted from the respective number of stimulated cells. **(D)** The pie charts show the proportion of single (grey), double (blue) and triple (yellow) and the bar chart the frequency (mean + SEM) of single, double and triple Th1 cytokine-producing CD4^+^ cells; the number of unstimulated cells was subtracted from the respective number of stimulated cells. Statistically significant differences to the antigen alone group were measured by two-way ANOVA with Dunnett’s post hoc test and are shown by circles (° p<0.05, °° p<0.01, °°° p<0.001, °°°° p<0.0001).

# Figure S2: Alternative mucosal route, cellular response.

Groups of 5 BALB/c mice were immunized sublingually either with PBS (control) or with two doses (21 days apart) of H5N1 virosomes (2.2 µg HA), alone or adjuvanted with c-di-AMP (5 µg), c-di-GMP (5 µg), CTB (10 µg) or LGA (7.5 µg). A control group received H5N1 virosomes (7.5 µg HA) + c-di-AMP (5 µg) intranasally. At 21 days after the second immunization, spleen cells were harvested, pooled and restimulated with homologous H5N1 virosomes. **(A) Cytokine production.** The number of cytokine-producing cells was determined by ELISpot. Results are presented as spot forming units of 10^6^ restimulated cells minus the unstimulated cell values. The mean + SD from triplicates in two cell concentrations are shown. Statistical significant differences were measured by one-way ANOVA with Tukey’s post hoc test and differences to the c-di-AMP or antigen alone group are shown by asterisks or circles, respectively (*/° p<0.05, **/°° p<0.01, ***/°°° p<0.001, ****/°°°° p<0.0001). **T cell quality.** At 21 days after the second immunization, spleen cells were harvested, restimulated with homologous H5N1 virosomes, intracellularly stained for the Th cytokines IFN-γ, IL-2, TNF-α, IL-4 and IL-17 and analyzed by flow cytometry. **(B)** Upper panel: All CD4^+^ cells producing at least one of the measured cytokines were summed to quantify the frequency of influenza-specific Th cells. The mean ± SD are shown with each symbol representing one animal. Lower panel: the mean + SEM of all CD4^+^ cells producing at least the indicated cytokine;, the number of unstimulated cells was subtracted from respective number of stimulated cells. **(C)** The frequency (mean + SEM) of single, double and triple Th1 cytokine producing CD4^+^ cells after subtraction of unstimulated cell values. Statistically significant differences were measured by two-way ANOVA with Tukey’s post hoc test and differences to the c-di-AMP group are shown by asterisks (* p<0.05, ** p<0.01, *** p<0.001, **** p<0.0001).

# Figure S3: CBA

Groups of 3 BALB/c mice were immunized intranasally either **(A)** with PBS (control) or with two doses (21 days apart) of H5N1 virosomes (7.5 µg HA), alone or adjuvanted with c-di-AMP (5 µg), c-di-GMP (5 µg), CTB (10 µg) or LGA (7.5 µg) or **(B)** with PBS (control) or with two doses (21 days apart) of H5N1 virosomes (7.5 µg, 2.5 µg, 0.5 µg or 0.1 µg HA), alone (only 7.5 µg HA) or adjuvanted with c-di-AMP (5 µg). At 21 days after the second immunization, spleen cells were harvested, pooled and restimulated with homologous H5N1 virosomes. The cytokine concentration was measured by CBA. Results from one representative experiment out of three are shown.

**Figure S4:** **Multifunctional T cells**

Gating of the CD3^+^8^+^ or CD3^+^CD4^+^ positive T cell population in a splenocyte sample derived from a vaccinated mouse showing **A)** the gating strategy for multifunctional T cells. As an example, CD3^+^CD4^+^ T cells producing TNFα with and without H5N1 stimulation were analyzed to quantify **B)** the frequency of TNFα secretion by influenza-specific Th cells.


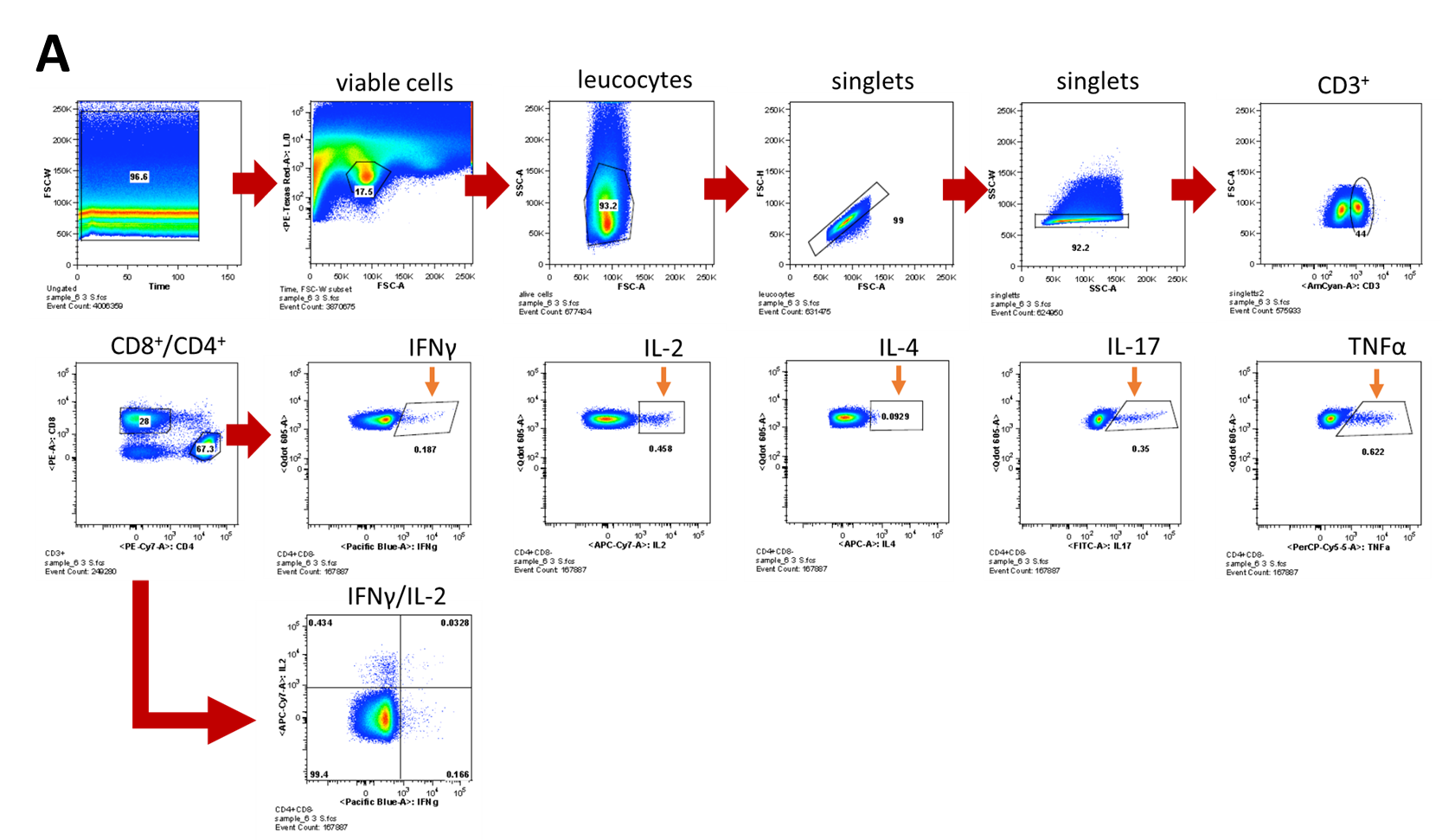


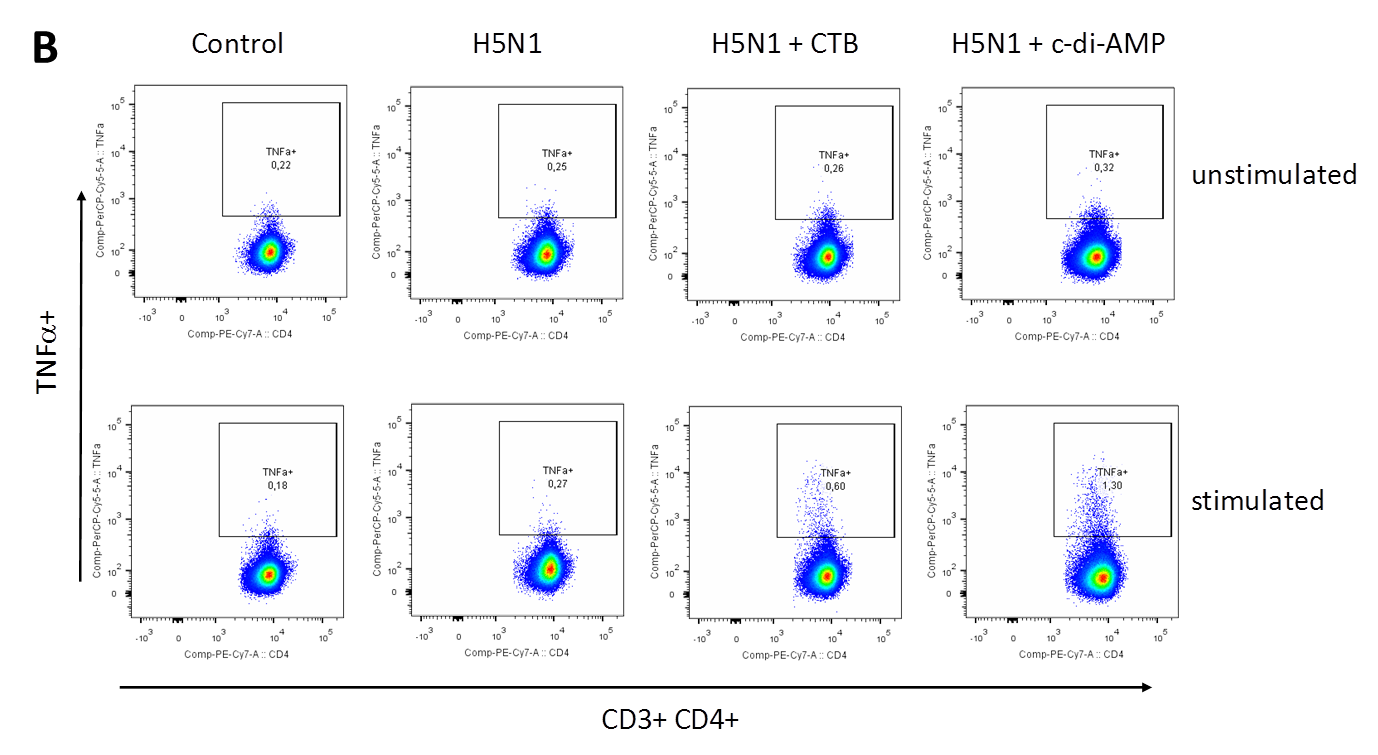

Supplement: Supplementary file 1 [file Data_Sheet_1.docx]
