# Supplementary material for: Mucosal Administration of Cycle-Di-Nucleotide-Adjuvanted Virosomes Efficiently Induces Protection against Influenza H5N1 in Mice
Source: Front Immunol. 2017 Sep 28;8:1223. doi: 10.3389/fimmu.2017.01223 (PMC5624999; doi:10.3389/fimmu.2017.01223)
Supplement: Supplementary file 2 [file Data_Sheet_2.docx]

# Supplementary Methods

## **Animals**

Six to eight week old female BALB/c mice were purchased from Harlan Winkelmann (Germany) or Janvier Labs (France) and maintained in the animal care facility of the Helmholtz Centre for Infection Research. All animal experiments were approved by and conducted in accordance to the regulations of the local government of Lower Saxony (Germany; No. 509.42502‑04‑017.08).

## **Immunization protocols**

Groups of mice (3-5 animals) were immunized either i.n. or s.l. on days 0 and 21 with PBS or isotonic saline (mock), or with H5N1 virosomes; the latter were administered alone or with different adjuvants – c-di-AMP, c-di-GMP, CTB (Sigma-Aldrich, Germany), last (or third) generation adjuvant (LGA) of immune stimulating complexes (Matrix M™) – made up to a maximal volume of 20 µl (i.n.) or 10 µl (s.l.) in PBS or isotonic saline solution. Matrix M™ (Isconova/Crucell) was produced from water extracts of *Quillaja saponaria* Molina bark, from which were derived two separately formulated saponin fractions A and C, mixed in defined proportions (91:9, respectively) before addition to the antigen (Pedersen et al. 2011). The ad-mix of antigen and adjuvant was performed 30 min before i.n. administration of 10 µl per nostril or s.l. administration of 8-10 µl. In order to standardize the immunization, the animals were temporarily anesthetized for maximally 30 min by i.p. injection of Ketamine/Rompun according to the manufacturer’s instructions (Inresa Arzneimittel GmbH, Germany and Bayer AG, Germany). The optimal amount of the adjuvant used was determined in previous studies (Ebensen et al. 2011).

## **Sample collection and processing**

Blood samples were collected on days 1, 20 and 42 via retro-bulbar bleeding. The samples were incubated for 60 min at 37°C and for 30 min at 4°C, then centrifuged to remove red blood cells (5 min at 8,000 × *g*), the resultant sera being stored at −20°C until processing. On day 42, saliva was collected prior to the blood sample by temporarily anesthetizing animals by i.p. injection of Ketamine/Rompun, with stimulation to salivate being achieved by i.p injection of pilocarpine hydrochloride (Sigma-Aldrich, Germany) at a concentration of 500 µg per kg mouse. After 5 to 10 min, saliva was collected (up to 200 µl) and stored at -20°C until processing. Then, mice were sacrificed and broncho-alveolar and nasal lavages were obtained by flushing the local tissues with ice-cold PBS supplemented with 5% (v/v) FCS (Greiner Bio-One, USA) and 40 µM phenyl-methane-sulfonyl-fluoride (PMSF). Lavages were centrifuged (5 min at 8,000 x *g*) to remove debris, then stored at -20°C. Cells were prepared from spleen and bone marrow to determine the presence of antigen-specific T and B lymphocytes (see below); the cellular responses were analyzed using pools of spleen cells, whereas antibody titers were assessed using individual animals, as previously described by (Borsutzky et al. 2006).

## **Virus and mouse challenge studies**

The influenza virosomal vaccine (Crucell, Netherlands) was produced from the reverse genetics-engineered seed virus (NIBRG-14), which was derived from a re-assortment between A/Vietnam/1194/2004 (H5N1) and A/Puerto Rico/8/34 (H1N1) (NIBSC, UK) (Mischler and Metcalfe 2002, Wood and Robertson 2004). These H5N1 virosomes contain the surface hemagglutinin (HA) and neuraminidase proteins embedded in a lipid membrane with no internal proteins. The influenza H5N1 NIBRG-14 virus was propagated in the chorio-allantoic cavity of 10-day-old embryonated hen eggs (Harlan Winkelman, Germany) for 48 h at 37°C (based on World Health Organization protocol (WHO 2002)) and used for serological assays and protective efficacy challenge experiments.

For challenge experiments, mice were anesthetized by i.p. injection with Ketamine/Rompun with doses adjusted to the individual body weight (e.g. 20 g/200 µl). Four weeks after the last immunization, 2 x 10^3^ focus forming units (ffu) of influenza H5N1 (A/Vietnam/1194/2004, NIBRG-14) virus were administered i.n. (20 µl in sterile PBS). Weight loss was followed up to 6 days post challenge. According to the regulations of the local government, animals showing more than 25% of body weight loss and peculiar behavior were euthanized.

## **Detection of antigen-specific IgG and IgA in serum**

The H5N1-specific antibodies were determined in serum samples by ELISA, using microtiter plates coated with 100 µl/well of the NIBRG-14 whole virus (2 µg/mL HA antigen in 0.05 M carbonate buffer, pH 9.6), as previously described for other antigens (Borsutzky et al. 2003). After overnight incubation at 4°C, plates were blocked with 3% (w/v) bovine serum albumin (BSA, Sigma-Chemie, Germany) in PBS for 1 h at 37°C. Serial two-fold dilutions of sera in 3% (w/v) BSA/PBS were added (100 µl/well), and plates were incubated for 2 h at 37°C. After washing with 1% (w/v) BSA/PBS/0.05% (v/v) Tween 20 (Carl-Roth, Germany), secondary antibodies were added – biotinylated goat anti-mouse IgA (Southern Biotechnologies, USA), biotinylated goat anti–mouse IgG (Sigma-Aldrich, Germany) or, to determine IgG subclasses, biotinylated goat anti–mouse IgG1, IgG2a and IgG3 (Sigma-Aldrich, Germany and Southern Biotechnologies, USA) – plates were further incubated for 2 h at 37°C. After washing, 100 µl of peroxidase-conjugated streptavidin (BD Bioscienes, Germany) was added to each well and plates were incubated at room temperature (RT) for 1 h. After another wash, reactions were developed using ABTS [2,20-azino-bis(3-ethylbenzthiazoline-6-sulfonic acid)] (Sigma-Aldrich, Germany) in 0.1M citrate-phosphate buffer (pH 4.35) containing 0.01% (v/v) H_2_O_2_. Endpoint titers were expressed as reciprocal values of the last dilution which gave an optical density at 405 nm of two times above the values of the negative controls. For calculation purposes negative samples were assigned an arbitrary titer of the lowest dilution measured.

## **Determination of antigen-specific IgA**

The amount of total and H5N1-specific IgA present in the lavages was determined by ELISA, as previously described for different antigens (Borsutzky et al. 2003). To compensate for variations among animals in the recovery efficiency of secretory antibodies, the results were normalized and expressed as endpoint titers of antigen-specific IgA per µg of total IgA present in the sample. For calculation purposes samples negative for specific IgA were assigned an arbitrary titer of the lowest dilution measured.

## **Determination of total IgE**

The content of total IgE in sera was determined using an anti-mouse IgE ELISA Kit (ELISA MAX™ Deluxe Set, BioLegend, USA) according to the manufacturer's instructions. Endpoint titers were expressed as absolute values of the last dilution which gave an optical density at 405 nm of two times above the values of the negative controls.

## **Measurement of cellular proliferation**

Spleens of vaccinated mice were aseptically removed, cell suspensions using pools of spleen cells of different immunized groups were prepared and erythrocytes were lysed (0.15 M NH_4_Cl, 1.0 M KHCO_3_, 0.1 mM EDTA, pH 7.2). Then, cells were washed twice and adjusted to 2 x 10^6^ cells/mL in complete RPMI medium (containing 10% (v/v) fetal bovine serum, 100 U/mL penicillin and 100 µg/mL streptomycin; Gibco, UK). Splenocytes were seeded at 100 µl/well (1 x 10^5^) in U-bottomed 96-well microtiter plates (Sarstedt Inc., Newton, NC) and cultured in quadruplicates for four days in the presence of different concentrations of inactivated NIBRG-14 virus with an HA concentration of 0.1-4 µg/mL; controls were 5 µg/mL concanavalin A or medium alone. Eighteen hours before harvesting, 1 µCi of [^3^H] thymidine (Amersham International, Freiburg, Germany) was added to each well. Cells were harvested on paper filters (Filtermat A; Wallac, Freiburg, Germany) using a cell harvester (Inotech, Wohlen, Switzerland). The incorporation of [^3^H] thymidine into the DNA of proliferating cells was determined using a scintillation counter (Wallac 1450, Micro-Trilux). The results are expressed as stimulation index (cpm stimulated/unstimulated).

## **ELISpot assay**

In order to determine the number of cytokine-secreting cells in the spleen, murine IFN-γ, IL-2, IL-4 and IL-17 ELISpot kits (BD Pharmingen) were used according to the manufacturer’s instructions. Spleen cells were incubated in two concentrations (2 to 4 x 10^5^/well) in triplicates for 16 h to 48 h in the absence or presence of inactivated NIBRG-14 virus with a final HA concentration of 0.2 µg/mL. Then, cells were removed and the plates processed according to manufacturer’s instructions. Colored spots were counted with an ELISpot reader (CTL-Europe GmbH) and analyzed using the ImmunoSpot image analyzer software v3.2.

For the detection of an induced long-lasting B cell response, antigen-specific B cells derived from the bone marrow were analyzed. Bone marrow-derived primary B cells were isolated by flushing the femur and tibia of the mice with medium, followed by erythrocyte lysis with ammonium chloride buffer ACK. Then, BM cells were washed and incubated in two concentrations (5 to 10 x 10^5^/well) in quadruplicates for 4 h on ELISpot plates coated either with inactivated virus (NIBRG-14) with a final HA concentration of 0.2 µg/mL or with a goat anti-mouse IgG capture antibody (Sigma-Aldrich, Germany) as control. After two washes with distilled H_2_O and three washes with PBS/0.1% (v/v) Tween, IgG producing cells were detected with a biotinylated goat anti-mouse IgG antibody (Sigma-Aldrich, Germany). Plates were further incubated overnight at 4°C. After three washes with PBS/0.1% (v/v) Tween, 100 µl of peroxidase-conjugated streptavidin (BD Bioscienes, Germany) was added to each well and plates were incubated at RT for 1 h. After another six washes, spots were developed for 5 to 60 min using AEC [3-Amino-9-ethylcarbazole] (Sigma-Aldrich, Germany) in 0.1 M acetate solution containing 0.01% (v/v) H_2_O_2_. Reactions were stopped using deionized water, plates were dried overnight at RT in the dark, and colored spots were counted with an ELISpot reader (C.T.L.) and analyzed using the ImmunoSpot image analyzer software v3.2.

## **Multiplex FlowCytomix (Cytometric bead array)**

In order to quantify the cytokines and chemokines secreted by splenocytes restimulated *ex vivo* using inactivated NIBRG-14 virus (NIBSC, UK) or H5N1 virosomes (Crucell, Netherlands) with an HA concentration of 0.1 up to 2 µg/mL, supernatants were collected on day 5 and stored at −70°C until processed. Then, the presence of mouse IL-1α, IL-2 , IL-4, IL-5, IL-6, IL-10, IL-13, IL-17A, IL-21, IL-22, IL-27, IFN-γ and TNF-α were determined in an immunoassay using a cytokine array according to the manufacturer's instructions (Mouse Th1/Th2/Th9/Th17 13plex FlowCytomix). In addition, chemokines or cytokines, such as IL-12, IL-18, RANTES, or GM-CSF were determined by the Mouse FlowCytomix Kit (Affymetrix eBioscience FlowCytomix Kit). Supplied standards were used to generate standard curves.

## **Serological assays**

HAI and MN assays were based on the World Health Organization protocols(WHO 2002).

## **HAI assay**

Sera were treated with receptor destroying enzyme (RDE, Sigma) by diluting one volume of serum in four volumes of RDE and subsequent overnight incubation at 37°C. RDE was then inactivated for 30 min at 56°C. Serial two-fold dilutions of RDE-treated sera starting from a 1:10 initial dilution in PBS were prepared in a V-bottom 96-well microtiter plate in a total volume of 25 µl each. Next, 8 hemagglutinating units of NIBRG-14 influenza virus were added to each of the serum dilutions. After 1 h, 25 µl of 1% (v/v) chicken erythrocyte suspension in PBS was added to each well of the plate and the plate was incubated at RT for another 30 min. After that the highest serum dilution able to inhibit hemagglutination was read from the plates. The reciprocal of this serum dilution represents the HAI titer of the respective serum. Negative samples were assigned an arbitrary titer of 5 for calculation purposes.

## **MN assay**

Madin-Darby Canine kidney (MDCK) cells seeded in 96-well microtiter plates at a density of 1.5 × 10^4^ cells per well in 100 µl Eagle’s Minimal Essential Medium (EMEM) supplemented with 10% (v/v) fetal bovine serum as previously described (WHO 2002). Mouse sera were treated with RDE (Sigma) as described above. Starting from a 1:10 initial dilution, a two-fold serial dilution of inactivated sera was set up in 50 µl volumes of virus diluent solution (DMEM containing 25 mM HEPES, 1% (w/v) BSA, 2.5 µg/mL NAT trypsin). Next, 100 TCID_50_ of the NIBRG-14 virus in 50 µl volumes of virus diluent solution were added to each of the serum dilutions, mixed gently and incubated for 1 h at 37°C, 5% (v/v) CO_2_. Following incubation, each virus-serum mixture was transferred to one of the MDCK cell monolayer wells of the plate (previously washed with the virus diluent solution without trypsin) and subsequently incubated overnight (18–22 h) at 37°C, 5% CO_2_. As controls, eight wells remained untreated (no virus infection, no antiserum addition – cell control) or were infected without addition of antiserum (virus control). After overnight incubation, cells were washed with PBS and fixed with ice-cold 80% (v/v) acetone (in PBS) for 10 min. Infection of cells was examined by ELISA detection of viral nucleoprotein (NP). Cells were washed three times with PBS containing 0.1% (v/v) Tween 20 and incubated with the primary antibody: Anti-influenza nucleocapsid polyclonal goat antibody (Virostat) diluted 1:1000, 100 µl/well in PBS with 1% (w/v) BSA and 0.1% (v/v) Tween 20 for 1 h at RT. Plates were washed three times before the second antibody anti-goat-HRP (KPL) was added (1:1000, 100 µl/well) for 1 h at RT. Plates were washed as above and 100 µl/well of freshly prepared O-phenylenediamine dihydrochloride substrate solution (10 mg in 20 mL phosphate/citrate buffer containing 0.03% (w/v) sodium perborate, Sigma–Aldrich) were added. After 5 min of incubation at RT, the reaction was stopped by addition of 100 µl/well of 0.5 N sulphuric acid. The absorbance (optical density, OD) was measured at 490 nm. The average absorbance of the virus control (VC) and the cell control (CC) were applied to calculate the virus neutralizing serum endpoint titer according to the following equation:

$$x=\frac{\left( Average OD of VC wells \right)- \left( Average OD of CC wells \right)}{2}+\left( Average OD of CC wells \right)$$

where *X* represents the 50% specific signal. All values below *X* were considered positive for neutralization and the reciprocal of the highest positive serum dilution was recorded as the neutralization titer of the respective serum. Negative samples were assigned an arbitrary titer of 50 for calculation purposes.

## **Cross-clade reactivity**

Cross-reactive immune responses against the drifted strains A/Cambodia/R0405050/2007 (clade 1.1), A/turkey/Turkey/1/2005 (clade 2.2.1) and A/Anhui/1/2005 (clade 2.3.4) were analyzed using the HAI assay.

## **Multifunctional T cells**

Splenocytes (2 x 10^7^ cells per well) were incubated (37°C, 5% CO_2_) in RPMI containing the HA antigen (NIBRG-14, 200 ng HA per mL) or no antigen to determine the basal cytokine production. After 16-20 h, 5 µg/mL brefeldin A (Sigma-Chemie, Germany) was added and the cells were incubated for an additional six hours. Subsequently, cells were stained for surface markers (CD3 and CD8, BD, USA; CD4, eBioscience, Germany) and dead cells (Fixable Dead Cell Stain, Invitrogen, USA), then fixed with 2% (w/v) *p*-formaldehyde (PFA), permeabilized for 45-60 min with 0.5% (w/v) Saponin in PBS/0.5% (w/v) BSA, and stained for intracellular cytokines (IL-2 and IFN-γ, BD, USA; IL-17, IL-4 and TNF-α, eBioscience, Germany). The gating strategy for the analysis of the multifunctional T cells is shown in Supplementary Figure S4 A. The cells were resuspended in PBS and light emission was measured using BD LSRII. After spectral overlap compensation with the BD FACS Diva Software, the data was analyzed using FlowJo (Tree Star, USA) on the basis of the following gating strategy: viable singlet leukocytes were gated for CD3^+^ CD4^+^ CD8^-^ and subsequently analyzed for the expression of IL-2, IL-4, IL-17, TNF-α (Supplementary Figure S4B) and IFN-γ.

## **Statistical analysis**

The statistical significance of the differences observed between the different experimental groups was analyzed using one-way ANOVA with Tukey’s or Dunnett’s post hoc test (GraphPad Prism v.6) with titers log_2_ normalized. Differences were considered significant at *p*< 0.05.

# References

Borsutzky, S., T. Ebensen, C. Link, P. D. Becker, V. Fiorelli, A. Cafaro, B. Ensoli, and C. A. Guzman. 2006. "Efficient systemic and mucosal responses against the HIV-1 Tat protein by prime/boost vaccination using the lipopeptide MALP-2 as adjuvant." *Vaccine* 24 (12):2049-56. doi: 10.1016/j.vaccine.2005.11.025.

Borsutzky, S., V. Fiorelli, T. Ebensen, A. Tripiciano, F. Rharbaoui, A. Scoglio, C. Link, F. Nappi, M. Morr, S. Butto, A. Cafaro, P. F. Muhlradt, B. Ensoli, and C. A. Guzman. 2003. "Efficient mucosal delivery of the HIV-1 Tat protein using the synthetic lipopeptide MALP-2 as adjuvant." *Eur J Immunol* 33 (6):1548-56. doi: 10.1002/eji.200323954.

Ebensen, T., R. Libanova, K. Schulze, T. Yevsa, M. Morr, and C. A. Guzman. 2011. "Bis-(3',5')-cyclic dimeric adenosine monophosphate: strong Th1/Th2/Th17 promoting mucosal adjuvant." *Vaccine* 29 (32):5210-20. doi: 10.1016/j.vaccine.2011.05.026.

Mischler, R., and I. C. Metcalfe. 2002. "Inflexal V a trivalent virosome subunit influenza vaccine: production." *Vaccine* 20 Suppl 5:B17-23.

Pedersen, G., D. Major, S. Roseby, J. Wood, A. S. Madhun, and R. J. Cox. 2011. "Matrix-M adjuvanted virosomal H5N1 vaccine confers protection against lethal viral challenge in a murine model." *Influenza Other Respir Viruses* 5 (6):426-37. doi: 10.1111/j.1750-2659.2011.00256.x.

WHO. 2002. WHO manual on animal influenza diagnosis and surveillance.

Wood, J. M., and J. S. Robertson. 2004. "From lethal virus to life-saving vaccine: developing inactivated vaccines for pandemic influenza." *Nat Rev Microbiol* 2 (10):842-7. doi: 10.1038/nrmicro979.
